# Anthropogenic legacies shaping the present composition of demarcation trees in a temperate upland field landscape in Japan

**DOI:** 10.1186/s13002-022-00543-7

**Published:** 2022-06-16

**Authors:** Tokuoka Yoshinori, Kimura Kenichiro, Oka Mitsunori

**Affiliations:** 1 Division of Biodiversity, Institute for Agro-Environmental Sciences, National Agriculture and Food Research Organization, 3-1-3, Kannondai, Tsukuba, Ibaraki 305-8604 Japan; 2 Rural Development Division, Japan International Research Center for Agricultural Sciences, 1-1, Ohwashi, sukuba, Ibaraki 305-8686 Japan; 3 Tokyo NODAI Research Institute, Tokyo University of Agriculture, 1-1-1, Sakuragaoka, Setagaya, Tokyo 156-8502 Japan

**Keywords:** Agro-biodiversity, Agricultural heritage, Ethnobotany, Historical ecology, Land-use change, Rural landscape

## Abstract

**Background:**

Isolated trees are often planted in agricultural landscapes around the world, but their planting background often remains unclear. In this study, we examined the history of demarcation trees in Ibaraki Prefecture in eastern Japan by using land dispute records mainly from the early modern period (from 1600 to 1868), the Rapid Survey Map (RSM) drawn in the late nineteenth century, demarcation tree records from 2011, and interviews of the local residents.

**Methods:**

We reviewed 39 documents on land disputes to examine the temporal and spatial usage of demarcation tree species in the early modern period. The association between the present distribution of 1486 individuals of six demarcation tree species and past land use in the RSM were analyzed with Fisher’s exact test and residual analysis. In addition, we conducted interviews with 48 farmers, most of whom were over 60 years old.

**Results:**

The demarcation plants in vast communal lands and village boundaries in the early modern period were mostly visually prominent tall trees, usually pines. In contrast, smaller trees were planted for demarcation in small-scale areas of forests and farmlands. Although *Pourthiaea villosa* (Thunb.) DC. Has been planted since the mid-eighteenth century, its planting seems to have accelerated as communal forests were divided mainly in the Meiji period (from 1868 to 1912). The present dominant state of *Deutzia crenata* Siebold et Zucc. in older farmlands and its ritual use, history of upland field development in the Kanto region, and ancient demarcation use in central Japan indicate its original use may date back to the medieval (from 1185 to 1600) or ancient *ritsuryo* period (from the seventh century to 1185). Tea (*Camellia sinensis* (L.) Kuntze) and mulberry (*Morus* spp.) individuals were considered as early modern or modern crop remnants. Results from the map-based analysis and interviews clarified the recent increase in the use of *Euonymus japonicus* Thunb. and *Celtis sinensis* Pers. for demarcation.

**Conclusions:**

Chronologically dynamic anthropogenic legacies have shaped the present agricultural landscape with different demarcation tree species. A better understanding of the dynamic transformation of vegetation under human influence adds to the historical heritage value of the landscape and should motivate its conservation.

**Supplementary Information:**

The online version contains supplementary material available at 10.1186/s13002-022-00543-7.

## Introduction

Mosaics of land uses in agricultural landscapes conserve biodiversity [1]. One important element supporting mosaics is a variety of vegetation maintained under human influence, including sacred groves, hedgerows, isolated trees, and farmlands under extensive management [2–4]. The aesthetic beauty of these kinds of vegetation is another important aspect contributing to improving people’s well-being [5–7]. Renes [8] expressed the concern that the history of these types of vegetation is often mischaracterized as being uniform. For example, recent research on the Amazonian landscape has suggested that the dynamics of plant use histories by ancient human groups determined current biodiversity patterns [9, 10]. To reconstruct the vegetation history, various types of evidence are available, for example, archaeological and botanical remains, literary records, old maps and photographs, written descriptions, and oral history [11]. However, the need for cross-disciplinary evaluation of information that is dispersed in various media and places has hindered research progress. Nonetheless, exploring the dynamics of non-crop vegetation under human influence is important to add heritage value, and motivate their conservation.

The dynamic history of European hedgerows has been well-documented [8]. The associations of plant composition and site types have been evaluated using multi-faceted information from archaeological features, written records (e.g., old census data), maps, photographs, plant distribution data, testimony of older people, and historical information on the origin of plants and chronological transformation of hedgerows [12]. The plant compositional characteristics of British hedgerows were intensively surveyed on a national scale [13]. Because of their historical value, the management, removal, and introduction of hedgerows has been regulated by the government [14, 15]. Trees other than hedgerows also contribute to shaping regionally unique agricultural landscapes, and the socio-economic and ecological importance of these trees has been evaluated in various countries [e.g., 16–18]. However, the evaluation of the planting history has often been limited to relatively short periods because there were often few materials available for verification.

During the *ritsuryo* period, which began in the seventh century, the imperial government of Japan adopted a land demarcation system using various features such as topographic landmarks and roads to outline the surrounding environment of a piece of land [19]. Such features indicating the surrounding environment were called *shiishi* and the local features identifying the boundary lines were called *bouji*; the local features were important for land demarcation at least since the medieval period, when land disputes over the private lands of nobles and temple and shrine territory had become common [19, 20]. Examples of such features in the *shiishi-bouji* system are the hedges, often called *kakiuchi, kaito, kaichi,* and *kakitsu*, that can be found in ancient and medieval documents [21, 22]. There is some folk nomenclature indicating that *Deutzia* was used for demarcation purposes, as well as for agricultural, medicinal, and ritual uses [23]. Toda [23] also pointed out the strong linkage between people’s religious beliefs during the *ritsuryo* period and the multiple functions of *Deutzia,* particularly *D. scabra* Thunb. var. *scabra.* The use of *D. crenata* for hedges in the gardens of nobles and peasants was also depicted in some Japanese *waka* (poetry) and essays in the ancient and medieval periods [24]. Although these fragmentary records provide a glimpse into the livelihoods of people at that time and help to partially reconstruct the historical landscape, details of the customs of peasants were seldomly recorded, including the use of demarcation trees in the upland field landscape.

Some of the demarcation trees in Japan were planted as hedges, but others were isolated or in the form of sparse hedges on boundary lines or at corners. Isolated planted trees in farmlands, generally known as *sakaigi*, are widely found in the Tohoku, Kanto, Chubu, and Shikoku regions in Japan [25]. Such use of isolated demarcation trees of *D. crenata* was recorded in Musashino in the Kanto region in the early modern period [26]. A novel by Nagatsuka [27] realistically describes many *D. crenata* in bloom in the southwestern part of Ibaraki Prefecture in the modern period. The spatial distribution patterns, folk nomenclature, and folk tool use of demarcation tree species were recently evaluated in several studies [28–30]. These studies indicated that the demarcation tree composition varied regionally and changed gradually over time. Species turnover was governed by various factors, such as ease of acquisition and transplanting, local horticultural or aesthetic preferences, historical trends of commercial crop and subsistence plant use, and a possible linkage with folk faiths. However, these studies primarily depend on oral histories, which constrained their ability to examine the dynamic history of demarcation tree transition across centuries.

In this study, in accordance with the history of land development, we hypothesized that different types of demarcation trees were introduced in each period, and that they remain as demarcation trees in upland fields to this day. To evaluate this hypothesis, we examined the following for Ibaraki Prefecture: (1) the relationships between demarcation tree species and land use recorded in land disputes between the seventeenth and nineteenth centuries, (2) the association between present-day demarcation tree distribution and past land use depicted in maps created in the 1880s, and (3) the results of interviews with older residents on local methods of defining forest demarcation and demarcation tree use.

## Materials and methods

### Land administration history in Japan

The following discussion of land administration in Japan from the ancient to early modern periods is based on [31]. In the ancient *ritsuryo* periods (from the seventh century to 1185), the imperial government of Japan did not have sufficient resources to directly rule over all of Japan, so the government delegated armed nobles known as samurai as local governors to keep public order and to collect rent in local manors. In the medieval period (from 1185 to 1600), the right to cultivate and the duties on a piece of land became complicated during the transition of various ruling structures such as the imperial legal and administrative system, manorialism, and the expansion of the privileges of samurai and farmers. In the late sixteenth century, Hideyoshi Toyotomi unified all of Japan and began a reform of the taxation system. In the reformed system, the holder of the right to cultivate a piece of land was obliged to pay tax only to a lord designated by Hideyoshi Toyotomi. However, under the rural self-governing system of taxation (*muraukesei*) in the early modern period (from 1600 to 1868), the use of various lands including shrub- and grasslands were actually managed by village communities and the taxes were paid collectively [32]. Private ownership and the sale of land were permitted, and land title certificates began to be issued by the Japanese government in 1872. In particular, during the modern Meiji period (1868–1912), many communal lands (commonly shrub- and grasslands) were divided and allocated to individuals from the government [33].

According to the Yoro Codes enacted in 757, there was a principle of public use of mountain resources such as grasses and shrubs (http://www.sol.dti.ne.jp/hiromi/kansei/yoro30a.html#09). Since the ancient *ritsuryo* period, grass was used as fertilizer in farmlands [34]. There are some records of conflicts over the use of mountain resources dating back to the medieval period [35]. In the early modern period, settlement of land disputes by violent force was prohibited by the Tokugawa Shogunate, and many written records of land dispute mediation at the shogunate and domain levels were collected in this study. In these documents, information such as the names of the villages involved in the dispute, vegetation conditions, the land-use history of the disputed area, and features indicating the land boundaries were included. Although we did not estimate the area of each piece of land disputed in this study, according to statistics on the area of communal land where classic land-use practices continued until the mid-1970s in the Kanto region, tens or hundreds of hectares of communal land were used by dozens to hundreds of community members in many communities [36].

## Demarcation practices around the early modern period

The municipal history materials for Ibaraki Prefecture and documents related to premodern history stored in the Ibaraki Prefectural Library were investigated. We selected 39 references by identifying reprints of explanations dealing with communal land disputes from their table of contents. Some of the references included a description of disputes over small-scale areas, such as farmlands, forests, and residences. Documents on land disputes in the early modern period sometimes included a map and a description of land demarcation conditions. It is often not feasible to carefully examine such maps without having high-quality copies of the originals or digitizing them and using georeferencing. In this study, we prioritized a broad overview of demarcation methods from the hundreds of dispute descriptions, and therefore omitted a careful interpretation of such maps. We believe that this approach is reasonable because the local species names of the demarcation trees, which is a main focus of this study, tended to be handled more clearly in the descriptions than in the maps.

**Fig. 1:**
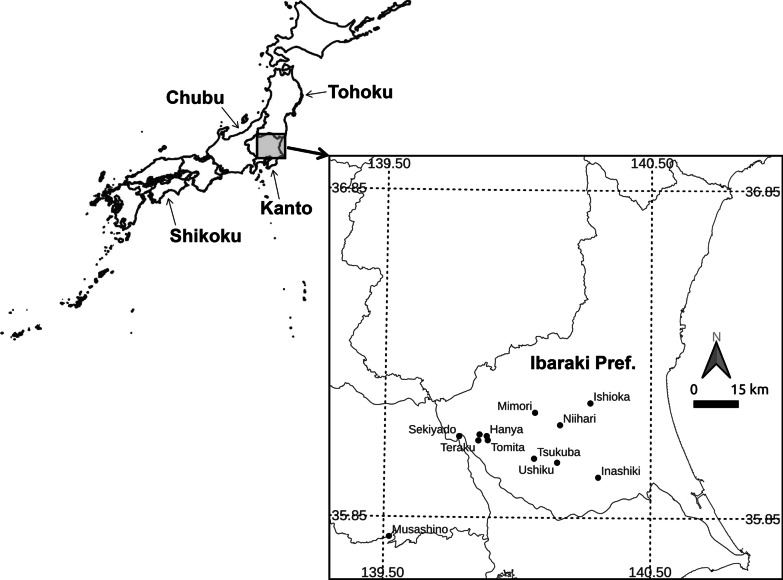
Maps of Japan and the Kanto region. The names of the locations were roughly positioned in the center of the area and municipalities

In this review of references, we collected the following information: the name of the village involved in each dispute, the latitude and longitude of the area mentioned which is roughly specified from the location of the villages by using Google Maps, demarcation methods, land-use where demarcation trees were planted, and the year in which each document was written. The locations of many of the places named in this study are shown in Fig. 1.

## Comparison of present demarcation tree location with nineteenth century land use

Data on the present distribution of demarcation trees in Ibaraki Prefecture were derived from the survey conducted by Tokuoka and Hosogi [28]. Plant nomenclature follows Yonekura and Kajita [37].

**Fig. 2:**
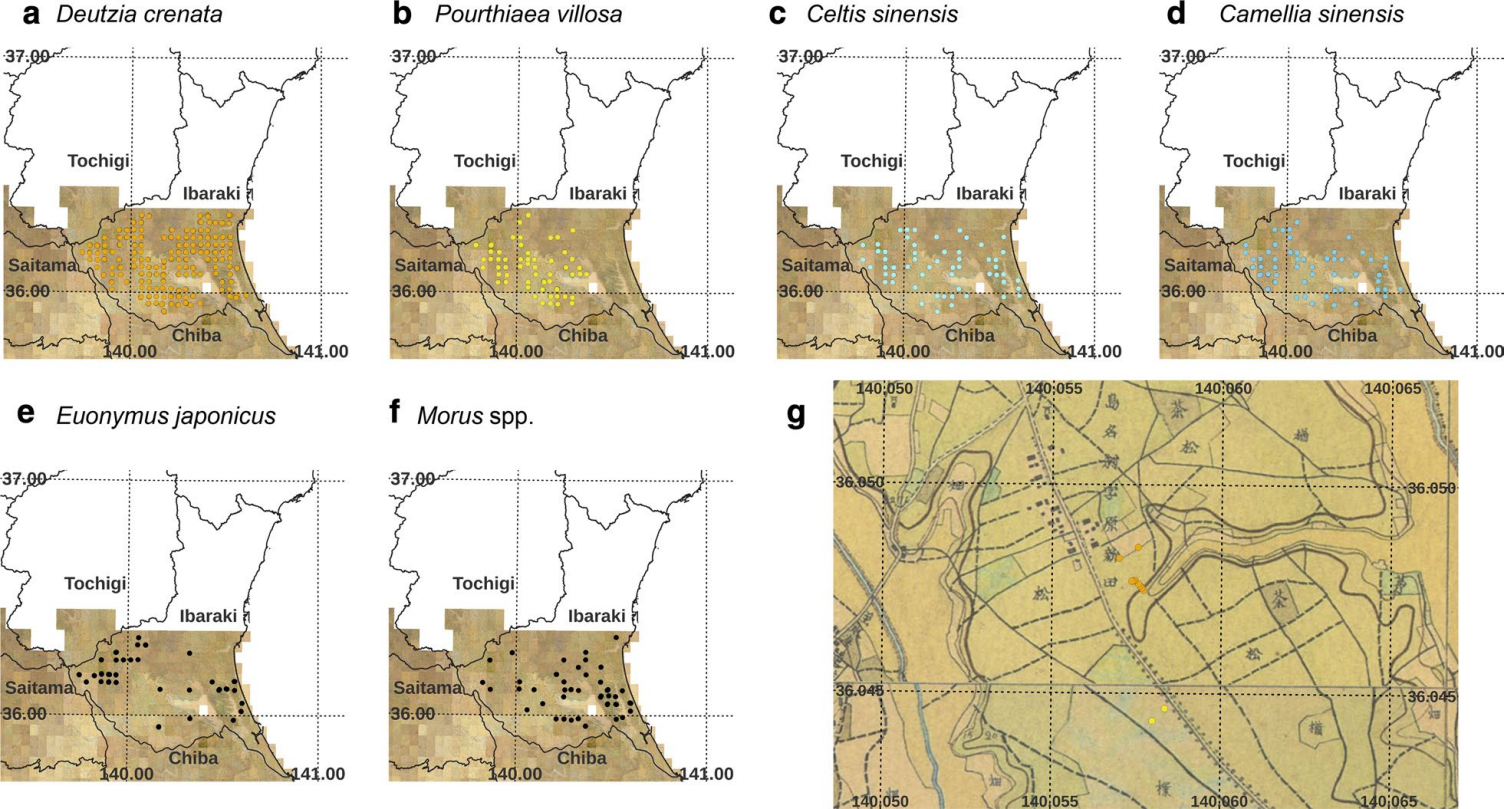
Overlay analysis of the association between demarcation tree location in 2011 and past land use in the 1880s in the Rapid Survey Map. Circles indicate the location of individual demarcation trees: **a** *Deutzia crenata* Siebold et Zucc., **b** *Pourthiaea villosa* (Thunb.) DC., **c** *Celtis sinensis* Pers., **d** *Camellia sinensis* (L.) Kuntze, **e** *Euonymus japonicus* Thunb., and **f** *Morus* spp. Areas in white were not mapped in the study (including the small square). **g** An example of a work screen for interpreting the land-use legend in the Rapid Survey Map of the planting position of each demarcation tree in Shimana village

The past land use of the planting position of the present demarcation tree individuals was interpreted by referring to the Rapid Survey Map (RSM) of the Kanto region that was illustrated between 1880 and 1886 on a scale of 1 to 20,000. The map was obtained from the web map service provided by the National Agriculture and Food Research Organization (aginfo.cgk.affrc.go.jp). Tree species with too few observed individuals were not suitable for analysis in statistical tests of the association between the planted location of present demarcation tree species and land use in the 1880s. Therefore, we selected six demarcation tree species for the analysis: *D. crenata* (*n* = 1085), *Pourthiaea villosa* (*n* = 165), *Camellia sinensis* (*n* = 128), *Euonymus japonicus* (*n* = 97), *Morus* ssp. (*n* = 87), and *Celtis sinensis* (*n* = 79). Tokuoka and Hosogi [28] adopted only one species name *Morus bombycis* based on the leaf morphology of the mulberries. However, according to some local farmers, some mulberry individuals left as demarcation trees seem to have been left after sericulture. According to statistics from 1915 [38], *M. alba* L. (*jumonji*) was most widely cultivated among the 49 varieties planted in Ibaraki Prefecture. Moreover, complex genetic background relationships have been noted for mulberry varieties used for sericulture [39]. Therefore, some of the *Morus* species used as farmland boundary trees might have been derived from artificially hybridized varieties, possibly including *Morus alba* and *Morus lhou* Koidz. as progenitors*.* Therefore, the general term *Morus* spp. was used in this study. Initially, we tried to interpret the past land use of all of the 1641 demarcation tree individuals of the six tree species (Fig. 2). Because of restrictions on map resolution, interpretation of the land use in the planted location of some trees was difficult. To minimize interpretation error, two authors (YT and KK) independently interpreted the land use of all 1641 individuals. The land-use interpretation was consistent for 1486 individuals, which were used for the subsequent analysis.

Land-use legends were interpreted depending on the Chinese characters or legend colors or both on the maps. Representative legends in the map were as follows: forests with specific species name or groups such as *Cryptomeria japonica* (L.f.) D.Don (*sugi*), *Quercus acutissima* Carruth. (*kunugi*), pines (*matsu*), deciduous *Quercus* (*nara*), coppices (species not specified, *zatsu* or *zou* and *ju*), paddy fields (*sui*), upland fields (*hatake*), tea plantations (*cha*), shrub-lands (*kan*, *are, boku, kou*), and grasslands (*kaya*, *kusa*). Vegetation conditions of shrub-lands and grasslands in the modern period are hard to distinguish [40]. Therefore, based on similar physiognomy or land-use purposes, these various land-cover types were simply categorized into three planting position types: farmlands (planting position was within paddy fields, upland fields, and tea plantations), forests (planting position was within land with tree name legends and coppice legends or the planting position was adjoining to them), and shrub- and grasslands (planting position was within shrub-lands, grasslands, or the planting position was adjoining to them). In the land-use interpretation that referred to the RSM and other map illustrations, Quantum GIS software version 3.20 [41] was used.

## Interviews on forest boundary demarcation practices

Semi-structured interviews were conducted with 48 respondents or groups of respondents in Ibaraki Prefecture, where we met them during our field survey on farmland demarcation trees from 3 March 2017 to 5 October 2021 (33 men, 13 women, and 2 couples). The age groups of the interviewees in the interviewed year were as follows: 40 s, *n* = 1; 60 s, *n* = 8; 70 s, *n* = 14; 80 s, *n* = 18; 90 s, *n* = 4, and unknown, *n* = 3. In each interview, the following predetermined items were discussed: demarcation methods of forest boundaries, local names of demarcation tree species, and whether the trees have uses other than demarcation. Additional information provided during the interview was also recorded.

## Statistical analysis

Fisher’s exact test (*P* < 0.05) was performed to test for associations between the locations of the six species and their past land uses in RSM. As a post-hoc test, residual analysis with the Holm correction (*P* < 0.05) was performed to test whether the occurrence of each species among three land-use types was significantly biased toward certain land uses. The statistical analysis was performed with R software version 3.6.3 [42].

## Results

### Demarcation practices around the early modern period

**Fig. 3:**
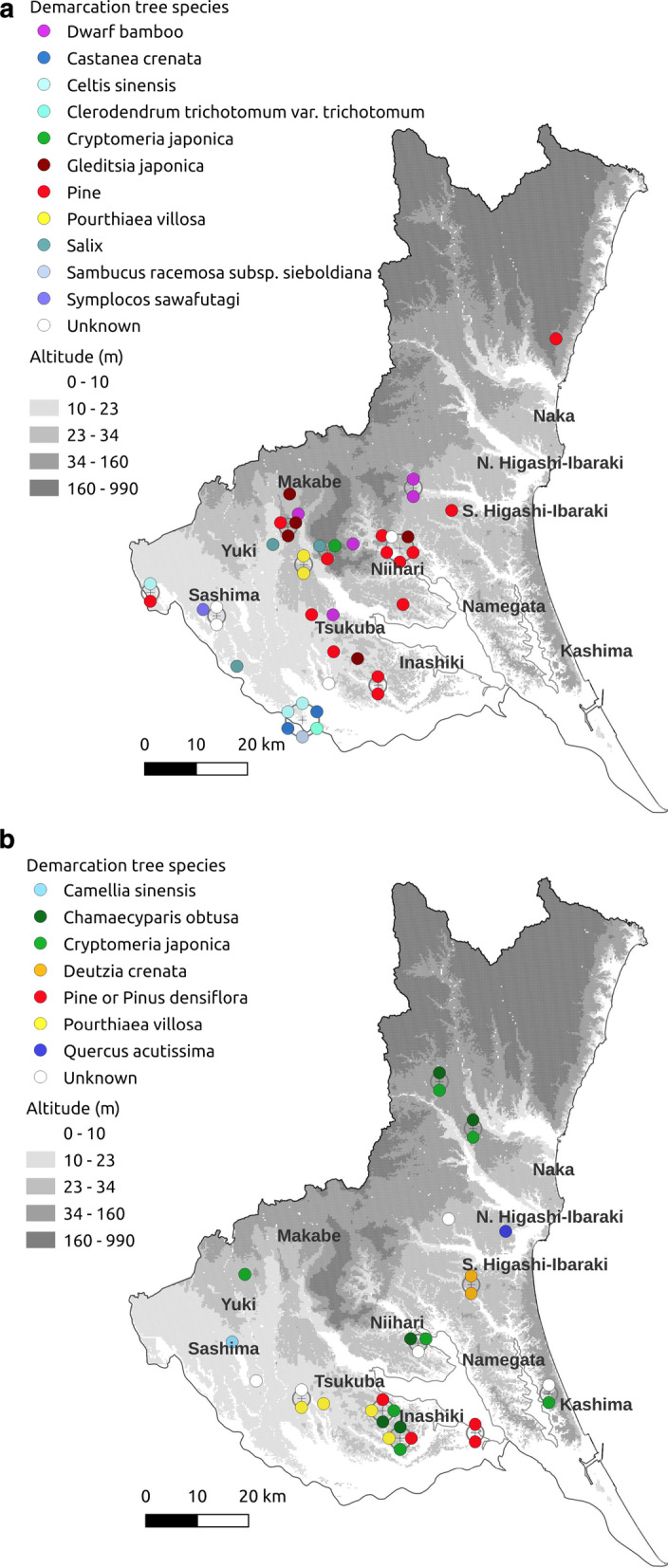
Demarcation plant distribution in Ibaraki Prefecture. **a** Species mentioned in documents written between 1648 and 1867 (*n* = 42), and **b** species mentioned by local farmers between 2017 and 2021 (*n* = 29). To avoid point overlap, the point displacement function was used to illustrate records having the same or similar longitude and latitude information. In such case, points were placed around a centroid (“ + ”)

In the 39 reference materials selected, we found 260 land demarcation methods used or mentioned between 1611 and 1876 in Hitachi Province and part of Shimousa Province (Additional file 1: Table S1). Artificially made objects such as mounds (*n* = 56), roads (*n* = 33), piles (*n* = 14), trenches (*n* = 14), and banks (*n* = 8) or topographic features such as ridges (*n* = 12) and streams (*n* = 9) were frequently mentioned. In total, 42 cases of demarcation plants or trees were found (Table 1, Fig. 3a). The demarcation plants in vast communal lands (*iriai*) and village borders were mostly visually prominent tall tree species such as pines (*n* = 14), *Gleditsia japonica* Miq. (*n* = 5)*,* and *Celtis sinensis* (*n* = 3). Pines and *C. japonica* were sometimes used as demarcation trees by row planting (Additional file 1: Table S1). In contrast, smaller trees such as *nishikori,* some of which were planted in a hedge form called *ikune* (*n* = 1), and *P. villosa* (*ushikoro(shi)*, *n* = 2) were planted for demarcation in relatively smaller forests and farmlands. In modern terms, *igune* (presumed to be similar to the word *ikune*) is a grove surrounding a farmhouse, and they are commonly found in the northern Kanto and Tohoku regions in Japan [43]. Referring to a note on folk nomenclature in the neighboring Tohoku region [44], which also has *igune*, we presumed that the species locally called *nishikori* corresponds to *Symplocos sawafutagi* Nagam*.*

**Table 1:** Demarcation plants or trees noted in reprints of documents of different types of disputed land boundaries between 1648 and 1867 in Hitachi Province and a part of Shimousa Province (present-day Ibaraki Prefecture)

Demarcation tree species or methods	Upland fields	Divided forest	Paddy fields	Buddhist invocation hall	Rental land (unknown land use)	Residence	Shrine	Border between upland fields and communal land	Border between upland fields and *goshuinchi* (temple-held grasslands)	Communal land	Village border	Total
*Pinus*	1	2								9	2	14
Dwarf bamboo										2	3	5
*Gleditsia japonica* Miq	1						1	1		2		5
*Celtis sinensis* Pers					1	1					1	3
Demarcation trees (unknown species)	2									1		3
*Salix*							1		1		1	3
*Castanea crenata* (L.f.) D.Don						1					1	2
*Pourthiaea villosa* (Thunb.) DC		1	1									2
*Clerodendrum trichotomum* Thunb. var. *trichotomum*						1						1
*Cryptomeria japonica* (L.f.) D.Don		1										1
*Sambucus racemosa* L. subsp. *Sieboldiana* (Miq.) H.Hara				1								1
Small tree planting (unknown species)	1											1
*Symplocos sawafutagi* Nagam	1											1

There were several examples of the use of demarcation trees. In documents written for Tomita and Hanya villages in 1675, officials of the domain ordered the use of demarcation trees by planting cuttings for upland field demarcation after reclamation of about 7 ha of forest lands. In a judgment letter from the Edo shogunate conference chamber in 1713 concerning a dispute involving three villages in the Ishioka region, an order was made to replace *G. japonica* planted for upland field demarcation with a small demarcation tree species because *G. japonica* interfered with rice production in the surrounding fields. According to an out-of-court settlement between Teraku and Oigo villages in 1775, about 5 ares of sparsely forested upland fields (*hayashibata*) were surrounded by a hedge (written as *ikune* in the document) made of *nishikori*, which was recognized as the demarcation structure. According to written records in 1852 (Additional file 1: Table S1), *P. villosa* was used for the demarcation of both forests and paddy fields in Mimori village. The reference also indicated that *P. villosa* had been planted in paddy fields since 1746. In a dispute in Shinchi village in 1867, demarcation trees of an unknown species were planted at the upland field boundary under the mediation of a temple priest.

## Comparison of present demarcation tree locations with the nineteenth century land-use records

There was a significant association between the planting locations of the six demarcation tree species and the past land uses (Fisher’s exact test, *P* < 0.001). Residual analysis showed that the planting position of *D. crenata* was positively biased toward farmlands and negatively biased toward forests and shrub- and grasslands in the modern period (Table 2). Similarly, the planting position of the tea plant *Cam. sinensis* was also positively biased toward farmlands and negatively biased toward shrub- and grasslands. In contrast, the positions of *P. villosa* and *Morus* ssp. were negatively biased toward farmlands and positively biased toward forests and shrub- and grasslands. The positions of *Cel. sinensis* and *E. japonicus* were not significantly biased toward any land use.

**Table 2:** Association between the location of six demarcation tree species recorded in 2011 and land use in the 1880s in the Rapid Survey Map

	Total number of individuals	Farmland		Forest		Shrub- and grassland	
		Number of individuals (%)	Standardized residual	Number of individuals (%)	Standardized residual	Number of individuals (%)	Standardized residual
*Deutzia crenata* Siebold et Zucc	985	615 (62.4)	5.3***	287 (29.1)	− 3.5**	83 (8.4)	− 3.3**
*Pourthiaea villosa* (Thunb.) DC	149	46 (30.9)	− 6.9***	68 (45.6)	3.7**	35 (23.5)	5.6***
*Camellia sinensis* (L.) Kuntze	120	85 (70.8)	3.1*	34 (28.3)	− 0.9	1 (0.8)	− 3.6**
*Euonymus japonicus* Thunb	88	48 (54.5)	− 0.6	29 (33.0)	0.1	11 (12.5)	0.7
*Celtis sinensis* Pers	74	35 (47.3)	− 1.8	32 (43.2)	2.1	7 (9.5)	− 0.2
*Morus* ssp.	70	26 (37.1)	− 3.5**	28 (40.0)	1.4	16 (22.9)	3.5**
Total	1486	855 (57.5)		478 (32.2)		153 (10.3)	

The standardized residual values labelled with asterisk(s) were significantly different from the expected values (* *P* < 0.05; ** *P* < 0.01; *** *P* < 0.001; residual analysis with the Holm correction)

## Interview results on the practices of forest boundary demarcation

Twenty out of 48 respondents or respondent groups mentioned the species names of demarcation trees or the planting method of demarcation trees in forests. The following demarcation tree species were mentioned (Fig. 3b): *C. japonica* (*n* = 7), *Chamaecyparis obtusa* (Siebold et Zucc.) Endl. (*n* = 5), unknown species (*n* = 5), *P. villosa* (*n* = 4), pine or *Pinus densiflora* Siebold et Zucc. (*n* = 4), *D. crenata* (*n* = 2), *Q. acutissima* (*n* = 1), and *Cam. sinensis* (*n* = 1). Some respondents indicated that demarcation trees were not planted directly on boundaries. Rather, they were planted in a row 90 cm (3 *shaku*, a unit of length used in the past) or 1 m away from the forest boundary line (*n* = 6), or different tree species were planted with existing species in an adjacent forest stand (*n* = 5). One interviewee from Tsukuba City called the practice of planting trees in a row 90–100 cm away from the boundary *herigi*. Another respondent said that the two owners planted tree species they had agreed upon.

In addition to demarcation tree use, other methods of identifying forest boundaries used characteristic landforms such as valleys (*n* = 3) and local features such as stones (*n* = 2), ditches (*n* = 2), mounds (*n* = 1), roads (*n* = 1), and charcoal (*n* = 1). A farmer in the Nakayama area in Tsukuba City stated that *P. villosa* individuals in the present upland fields were originally maintained as demarcation trees in forests, and they were left undisturbed after the land was converted to farmland soon after WWII. Another respondent from the Midorino area in Tsukuba City also stated that *P. villosa* was used as demarcation trees in forests.

Other than those reported in the previous study [28], there are several additional information. The local names of *P. villosa* were* ushikoroshi* (*n* = 4) or *ushikoro* (*n* = 2). It was used as materials for handles such as hand axes (*n* = 1), hoes (*n* = 1), sickles (*n* = 1), hammers (*n* = 1) and for saddles of cows (*n* = 1). At the time of the WWII, *D. crenata* were used as materials for wooden nails needed for paulownia chests (*n* = 1).

A few respondents noted changes in demarcation tree species in upland fields. For example, one said that *D. crenata* was older than *Cel. sinensis* and two said it was older than *E. japonicus* (*n* = 2). An interviewee in the Inashiki region stated that *E. japonicus* individuals may be derived from the hedges of a surrounding residence.

## Discussion

### Demarcation practices in vast areas

As shown in Table 1, in the early modern period, pine was the representative demarcation species, followed by *G. japonica* and *Cel. sinensis*. These tall tree species could easily be seen by many village members in vast communal lands and as village boundaries. Visible tall trees were commonly planted in vast farmlands [17], under various land-use conditions [12], and as milestone trees [45]. The ramets of dwarf bamboos spread laterally in soils, however, and it is unlikely that bamboo was planted at the land boundary as a permanent demarcation plant. Therefore, we presume that it was temporarily used as a substitute for piles as a guideline for use during an arbitration period.

As shown in Additional file 1: Table S1 and our interview results, mounds, roads, banks, signboards, stakes, and natural topographic features such as peaks and rivers appear to have been commonly used for demarcation methods. These non-tree features were also highly visible to many people. Moreover, if they were not intentionally destroyed, many of these structures were physically quite strong and could be maintained for a long period of time between generations.

The practice of *herigi* and planting of different afforestation trees species in adjacent forests, whose planting positions were away from the boundary lines, indicates that the local people perceived that boundary lines of forests were less necessary in forested areas than on farmland parcels.

These results indicate that the land demarcation system for vast areas used quite different methods and underlying concepts than those used in small-scale areas like upland fields, which relied heavily on small trimmed tree plants.

## Possible use of *Deutzia ﻿crenata* since the ancient period

In the study area, *D. crenata* was the dominant demarcation tree; it accounted for 60.7% of surveyed trees of upland fields [28]. As shown in Table 2, this species was significantly abundant in places having an older farmland legacy. In the early modern period, *D. crenata* was used for demarcation in the Musashino region, which is close to our study area [26]. Many of the elders interviewed were born in the first half of the twentieth century and said that most demarcation tree individuals had been inherited from their ancestors and had been maintained without replanting [28]. In the study area, *D. crenata* has been associated with folklore use, including as a walking stick in burial outfits [28] and as a bow in the Obisha Ritual [46]. Moreover, it was also used for wooden nails in the production of *Paulownia* cabinets. Although multiple records document the custom of using demarcation trees and hedges to express land demarcation in the early modern period, a major remaining research challenge is the assessment of how far back from the early modern period this practice dates.

Based on the Kofun (ancient graves) distribution, it is conjectured that villages and upland fields were widely spread in the plateau area of the Kanto region in the third to seventh century [47]. In *Wamyosho* (https://dl.ndl.go.jp/info:ndljp/pid/2544216), which was written in the tenth century, present-day place names such as Niihari, Inashiki, and Tsukuba were written. By that time, there were already people living on these plateaus, and upland field cultivation would have advanced to a certain extent. A major obstacle to reconstructing the past rural landscape is the paucity of medieval documents. However, the development of upland fields described in the cadastral surveys in the early modern period can be interpreted as having a certain degree of continuity of development from the medieval period [47]. According to Farris [48], Japan’s total arable area was estimated to have increased by 1.66 times from 1150 to 1600. If these estimates are reasonable and applicable to our study area, a significant portion of the upland fields in the early modern period have been under cultivation since the medieval period. In the medieval period, development of upland fields was conducted by establishing hedges around upland fields [22], similar to use of hedges for land demarcation in the ancient period [23].

Our RSM results (Table 2), the documentation of *D. crenata* use since the early modern period in the Kanto region, the general pattern of rural development coinciding with the use of a demarcation system since the ancient period, and the present dominant state of *D. crenata* use for present upland field demarcation all suggest that both hedges and isolated trees (mainly *D. crenata*) were adopted for use as a demarcation system of upland fields in the study area and its vicinity from the ancient or medieval period.

## *Pourthiaea villosa* mainly planted for divided forests

Two of the interviewees from Tsukuba City indicated the significant linkage of *P. villosa* with forests. Moreover, a folklore note [49] showed that, by taking advantage of the soft nature of *P. villosa* branches, people could tie a branch into a circle so that it was visible as a demarcation mark in the forests of Ushiku City, which is adjacent to Tsukuba City. In our RSM analysis, *P. villosa* was significantly abundant both in former forests and in shrub- and grasslands (Table 2). At present, vast shrub- or grassland areas are rare in the study region, so the oral history of species use obtained in the folklore note and our interviews may indicate the species use in forests. Information on folk tools also implied that the species’ usefulness was one of the favored reasons to use the species for demarcation. In Japan, land parcel allocation to individuals by *wariyama* (divided forests) was found to occur from the fourteenth to twentieth centuries [50], but the practice mainly spread in the modern Meiji period when the ownership of communal lands was allocated to individuals [33]. Many of the shrub- and grasslands in the RSM were vast in area, and geometrically shaped upland fields were sparsely distributed within them. Such land-use patterns indicate that land allocation by *wariyama* was occurring at that time. Although the demarcation use of *P. villosa* has certainly been practiced since at least 1746, in light of the history of *wariyama* expansion and local people’s perceptions of its use in forests, we presume that the planting of *P. villosa* for demarcation purposes accelerated at the beginning of the modern period.

**Fig. 4:**
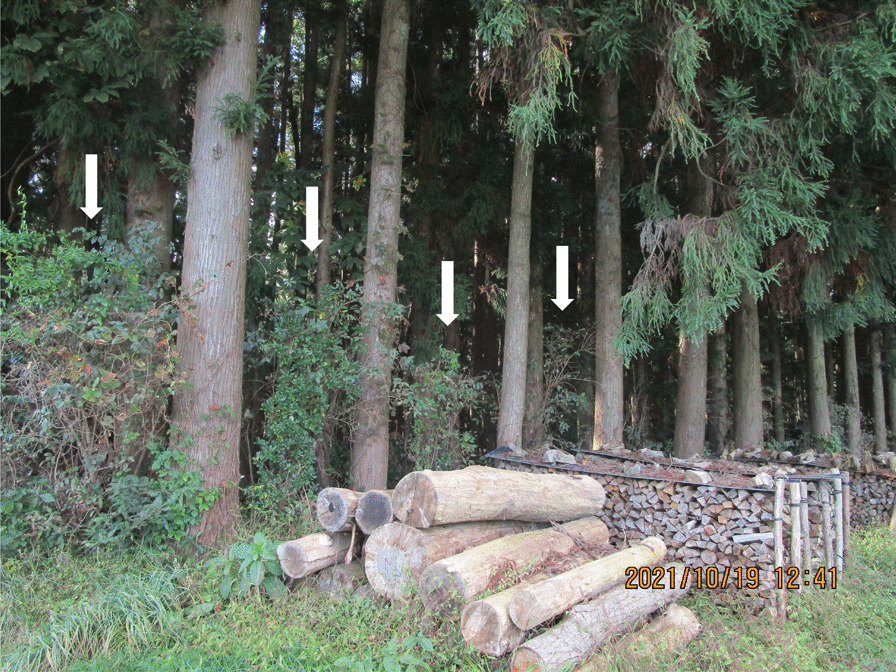
Examples of *Deutzia crenata* Siebold et Zucc. planted for forest demarcation in Aomori Prefecture, northern Japan. Individual *D. crenata* trees (marked with white arrows) were planted between trunks of *Cyptomeria japonica* (L.f.) D.Don at the edge of a forest parcel adjacent to farmland

On the other hand, in the central part of the prefecture, where *P. villosa* was not used for demarcation, *D. crenata* was said to be used for demarcation in forests. Although it is located far north of our study area, one of the authors (YT) confirmed in 2021 that a row planting of *D. crenata* was maintained at a boundary between a small-scale artificial forest and an upland field in Aomori Prefecture in the Tohoku region of northeastern Japan (Fig. 4). These results imply that the two species are physiologically tolerant for use as demarcation trees in small-scale areas with different types of above-ground vegetation, such as upland fields and artificial forests. Moreover, the regionally preferred demarcation tree species—*D. crenata* in the central part and *P. villosa* in the south and southwestern parts of Ibaraki Prefecture—differed as the division of communal land progressed during the modern period.

## Crop remnants: tea and mulberry

In the study area, it is said that the first tea introduction dated back to the fourteenth century, but tea is commonly recorded in the land register in the early seventeenth century [51]. In 1665, the lord of the Shimousa Sekiyado domain in the western part of the study area ordered that tea be planted on upland field boundaries [51]. According to statistics of the modern period [52], tea production (in terms of area planted) in Ibaraki Prefecture was stably high at about 2000 to 3000 ha during 1880s to 1920s. These facts and the positively biased planting of tea plants toward farmlands (Table 2) together imply that demarcation *Cam. sinensis* trees have their origin in the proliferation of tea plants on upland field boundaries in the early modern period.

The area of mulberry leaf production was quite small in the late nineteenth century in Ibaraki Prefecture, only about 692 ha in 1883 [52]. Its production continued to increase and reached its peak around the 1930s when the production area was about 27,000 ha. In the Namegata area, mulberry trees were planted in the upland fields reclaimed from communal lands in the modern period [53]. This report corresponds well to our finding that mulberry was abundant in the shrub- and grasslands in the RSM (Table 2), which possibly were mostly former communal lands. Former tree crops were left at upland field boundaries in other regions, for example, *Salix koriyanagi* Kimura ex Goerz (used for making baskets and furniture) and mulberry (used for sericulture in Shikoku) [29, 30]. These results indicate that some individuals of woody crop species tended to be left behind as demarcation trees after their production had peaked.

## Recent increase in *Euonymus japonicus* and *Celtis sinensis*

The planting position of two species, *E. japonicus* and *Cel. sinensis*, was not significantly biased toward either land use in RSM, suggesting little influence of past land use on the presence of the two species in the present upland field demarcation. The planting of *E. japonicus* for garden plant use increased soon after WWII in some regions in the Kanto and Shikoku regions [26, 29]. Some of our interviewees’ comments on demarcation tree changes and their origin implied that *Cel. sinensis* and *E. japonicus* may have been introduced relatively recently, and the latter were derived from hedges around residences. These conditions imply that the garden plant proliferation after WWII may be a common reason for the proliferation of *E. japonicus* for use as demarcation trees*.* No interviewees during the current or previous interviews in 2011﻿ [28] mentioned traditional uses of *Cel. sinensis* other than as demarcation trees. Although *Cel. sinensis* grows tall, it may have been chosen for use in demarcation because of its sprouting ability in the face of repeated trimming in upland fields. The introduction history of this species should be examined in future studies.

## Conclusions

The present study elucidated the multi-faceted anthropogenic legacies influencing current biodiversity patterns. The methodological effectiveness of our research approach, which combines socio-economic, past land use, and ethnobotanical information, was demonstrated in historical ecology, and it can be applied in a similar manner to elucidate the dynamics of vegetation under human influences during recent centuries in various agricultural landscapes. We confirmed that demarcation trees in the Kanto region existed steadily from the early modern period, and their origin may date back to much earlier times. The progress of *wariyama* forest dividing, shifting crop species, and the trends in the use of garden plants influenced the reasons various demarcation tree species were introduced. These chronologically dynamic anthropogenic legacies have shaped the present agricultural landscape with different demarcation tree species. Additional study is necessary to establish methodologies to explore the still unclear use of demarcation trees in the medieval and ancient periods. Moreover, the history of the use of demarcation trees in other regions should also be evaluated to identify its origin, cultural transformation, and regional variation. Although many demarcation tree individuals have been preserved in many places, a number of them are being removed under agricultural intensification. Therefore, efforts must be made to evaluate their history and the various ecosystem services that they provide and to conserve them.

## Supplementary Information

**Additional file 1:** Information on the demarcation methods used or mentioned between 1611 and 1876 (*n *= 260) in the 39 references.

## Data Availability

All of the data supporting the conclusions of this article are included within the article.
