# Supplementary material for: Anthropogenic legacies shaping the present composition of demarcation trees in a temperate upland field landscape in Japan
Source: J Ethnobiol Ethnomed. 2022 Jun 16;18:45. doi: 10.1186/s13002-022-00543-7 (PMC9202135; doi:10.1186/s13002-022-00543-7)
Supplement: Supplementary file 1 — Additional file 1: Information on the demarcation methods used or mentioned between 1611 and 1876 (n = 260) in the 39 references. [file 13002_2022_543_MOESM1_ESM.doc]

**Additional file 1 Information on the demarcation methods used or mentioned between 1611 and 1876 (*n* = 260) in the 39 references.**

| References | Village disputed | Latitude  (north) | Longitude  (east) | Demarcation method | Land use where demarcation trees were planted | Year |
| --- | --- | --- | --- | --- | --- | --- |
| Yasato-choushi-hensan-iinkai. Yasato-choushi. Ibaraki: Elite Insatsu; 2005. (in Japanese) | Fuchu_Shimobayashi_Katano_Kanasashi | 36.222019 | 140.232859 | Pine | Communal land | 1648 |
| Ishioka-shishi-hensan-iinkai. Ishioka-shishi-gekan. Tokyo: Daiichihouki; 1985. (in Japanese) | Fuchu_Yamazaki | 36.200198 | 140.271943 | Pine | Communal land | 1648 |
| Akeno-choushi-hennsanniinkai. Akeno-choushi-shiryou dai21shuu kinseishiryou6 Akeno-no-soshoudeiri1. Fukuoka: Saneiinsatsu; 1994. (in Japanese) | Ebigashima_Kuwayama | 36.29332 | 140.0324 | *Gleditsia japonica* Miq. | Shrine | 1649 |
| Yasato-choushi-hensan-iinkai. Yasato-choushi. Ibaraki: Elite Insatsu; 2005. (in Japanese) | Shimoaoyagi_Tsukioka | 36.206986 | 140.170416 | Erect a dwarf bamboo | Communal land | 1650 |
| Iwamamachi-kyouikuiinkai. Iwama-choushi. Ibaraki: Iwamamachi; 2002. (in Japanese) | Oogoyama_Haji | 36.312079 | 140.301384 | Insert a dwarf bamboo | Village border | 1656 |
| Iwamamachi-kyouikuiinkai. Iwama-choushi. Ibaraki: Iwamamachi; 2002. (in Japanese) | Iwama_Oogoyama_Haji | 36.30009 | 140.29896 | Erect a dwarf bamboo | Village border | 1656 |
| Ishioka-shishi-hensan-iinkai. Ishioka-shishi-gekan. Tokyo: Daiichihouki; 1985. (in Japanese) | Fuchu_Murakami_Someya | 36.200198 | 140.271943 | Pine | Communal land | 1664 |
| Goka-choushi-hensaniinkai. Choushi-Goka-no-seikatsushi shiryou1. Tokyo: Gyousei; 2011. (in Japanese) | Motokurihashi_Shinkouya | 36.114046 | 139.739754 | Pine (probably on both sides of the boundaries) | Village border | 1670 |
| Iwaishishi-hennsanniinkai. Iwaishishi-shiryou-kinseihen2. Tokyo: Daiichihouki; 1995. (in Japanese) | Tomita_Hanya | 36.089486 | 139.887802 | Demarcation trees | Upland fields | 1675 |
| Yasato-choushi-hensan-iinkai. Yasato-choushi. Ibaraki: Elite Insatsu; 2005. (in Japanese) | Kohata_Tsukuba | 36.26687 | 140.38336 | Pine | Communal land | 1675 |
| Tsuchiurashi-kyouikuiinkai. Tsuchiurashi-bikou-dai5kan. Ibaraki: Tsuchiurashi-kyouikuiinkai; 1996. (in Japanese) | Sakemaru_Omonoi_Karima_Hiratsuka | 36.082572 | 140.08232 | Line planting of pines | Communal land | 1688 |
| Tsuchiurashi-kyouikuiinkai. Tsuchiurashi-bikou-dai5kan. Ibaraki: Tsuchiurashi-kyouikuiinkai; 1996. (in Japanese) | Kurakake_Hanamuro_Onozaki | 36.082663 | 140.128445 | Erect a dwarf bamboo | Communal land | 1688 |
| Tsuchiurashishi-hennsanniinkai. Tsuchiurashishi-hennshuushiryou-dai12hen. Ibaraki: Inamoto-Insatsusho; 1970. (in Japanese) | Migimomi_Karasuyama_Arakawa_Jitsukoku | 36.006527 | 140.182211 | ***Saitsuchi***? (misspelling of ***saikachi***?: *Gleditsia japonica*) | Communal land | 1689 |
| Akeno-choushi-hennsanniinkai. Akeno-choushi-shiryou dai21shuu kinseishiryou6 Akeno-no-soshoudeiri1. Fukuoka: Saneiinsatsu; 1994. (in Japanese) | Miyayama_Miyago | 36.25841 | 140.05148 | Erect a dwarf bamboo | Village border | 1693 |
| Kukizaki-choushi-hennsanniinkai. Kukizaki-choushi-hennsanniinkai-shiryou-kinsei-kindaishi-shiryoushu. Ibaraki: Isebu; 1993. (in Japanese) | Ooi_Hinosawa | 36.018082 | 140.130507 | Pine | Communal land | 1700 |
| Akeno-choushi-hennsanniinkai. Akeno-choushi-shiryou dai21shuu kinseishiryou6 Akeno-no-soshoudeiri1. Fukuoka: Saneiinsatsu; 1994. (in Japanese) | Kuramochi_Teraueno | 36.235362 | 140.02982 | *Gleditsia japonica* (old demarcation tree) | Border between upland fields and communal land | 1702 |
| Akeno-choushi-hennsanniinkai. Akeno-choushi-shiryou dai21shuu kinseishiryou6 Akeno-no-soshoudeiri1. Fukuoka: Saneiinsatsu; 1994. (in Japanese) | Kuramochi_Teraueno | 36.235362 | 140.02982 | Line planting of *Gleditsia japonica* | Communal land | 1702 |
| Akeno-choushi-hennsanniinkai. Akeno-choushi-shiryou dai21shuu kinseishiryou6 Akeno-no-soshoudeiri1. Fukuoka: Saneiinsatsu; 1994. (in Japanese) | Kuramochi_Teraueno | 36.235362 | 140.02982 | Line planting of pines | Communal land | 1702 |
| Ishioka-shishi-hensan-iinkai. Ishioka-shishi-gekan. Tokyo: Daiichihouki; 1985. (in Japanese) | Fuchu_Murakami_Someya | 36.200198 | 140.271943 | ***Saikara***? planting (misspelling of ***saikachi***?: *Gleditsia japonica*) | Upland fields | 1713 |
| Ishioka-shishi-hensan-iinkai. Ishioka-shishi-gekan. Tokyo: Daiichihouki; 1985. (in Japanese) | Fuchu_Murakami_Someya | 36.200198 | 140.271943 | Small tree planting | Upland fields | 1713 |
| Ishioka-shishi-hensan-iinkai. Ishioka-shishi-gekan. Tokyo: Daiichihouki; 1985. (in Japanese) | Fuchu_Murakami_Someya | 36.200198 | 140.271943 | Pine | Village border | 1713 |
| Sakai-choushi-hensaniinkai. Shimousasakai-no-seikatsushi shiryouhen kinseiII mura-no-seikatsu. Maebashi: Asahiinsatsu; 2002. (in Japanese) | Kamiizushima | 36.06814 | 139.867649 | Demarcation trees | Communal land | 1713 |
| Ushikushi. Ushikushi-shiryou-kinseiII-muratoseikatsu. Tokyo: Seikousha; 1997. (in Japanese) | Kuno_Kosaka_Shoujiki_Shimada | 35.959603 | 140.226418 | Pine | Upland fields | 1740 |
| Ushikushi. Ushikushi-shiryou-kinseiII-muratoseikatsu. Tokyo: Seikousha; 1997. (in Japanese) | Kuno_Kosaka_Shoujiki_Shimada | 35.959603 | 140.226418 | Mound and pine | Communal land | 1740 |
| Sekijyou-choushi-hennsanniinkai. Sekijyou-choushi-shiryouhen1. Tokyo: Seikousha; 1983. (in Japanese) | Takasai | 36.20465 | 139.997346 | *Salix* sp. | Border between upland fields and goshuinchi (temple held grasslands) | 1748 |
| Goka-choushi-hensaniinkai. Choushi-Goka-no-seikatsushi shiryou1. Tokyo: Gyousei; 2011. (in Japanese) | Kawatsuma_Motokurihashi | 36.122159 | 139.727275 | *Celtis sinensis* Pers. | Village border | 1753 |
| Sashimachoushi-hennsanniinkai. Sashimachoushi-shiryouhen-kinsei. Tokyo: Seikousha; 1995. (in Japanese) | Teraku_Oigo | 36.089627 | 139.847965 | Hedge (***nishikori***; presumed to be *Symplocos sawafutagi* Nagam. by referring to Yasaka Shobou. Nihon-shokubutu-hougen-shusei. [Collection of Japanese plant dialects]. Yasaka Shobou; 2001. (in Japanese). | Upland fields | 1775 |
| Iwaishishi-hennsanniinkai. Iwaishishi-shiryou-kinseihen2. Tokyo: Daiichihouki; 1995. (in Japanese) | Sugao_Houshito_Yahagi | 35.990922 | 139.922622 | *Salix* sp. | Village border | 1778 |
| Torideshishi-hennsanniinkai. Torideshishi-kinseishiryou3. Tokyo: Seikousha; 1991. (in Japanese) | Toride_Daishuku | 35.897361 | 140.064595 | Demarcation tree (*Sambucus racemosa* L. subsp. *sieboldiana* (Miq.) H.Hara) | Buddhist invocation hall | 1814 |
| Torideshishi-hennsanniinkai. Torideshishi-kinseishiryou3. Tokyo: Seikousha; 1991. (in Japanese) | Toride_Daishuku | 35.897361 | 140.064595 | Demarcation tree (*Clerodendrum trichotomum* Thunb. var. *trichotomum*) | Residence | 1814 |
| Torideshishi-hennsanniinkai. Torideshishi-kinseishiryou3. Tokyo: Seikousha; 1991. (in Japanese) | Toride_Daishuku | 35.897361 | 140.064595 | Demarcation tree (*Castanea crenata* Siebold et Zucc.) | Residence | 1814 |
| Torideshishi-hennsanniinkai. Torideshishi-kinseishiryou3. Tokyo: Seikousha; 1991. (in Japanese) | Toride_Daishuku | 35.897361 | 140.064595 | Demarcation tree (*Celtis sinensis*) | Rental land (unknown land use) | 1814 |
| Torideshishi-hennsanniinkai. Torideshishi-kinseishiryou3. Tokyo: Seikousha; 1991. (in Japanese) | Toride_Daishuku | 35.897361 | 140.064595 | Stump (*Celtis sinensis*) | Residence | 1814 |
| Torideshishi-hennsanniinkai. Torideshishi-kinseishiryou3. Tokyo: Seikousha; 1991. (in Japanese) | Toride_Daishuku | 35.897361 | 140.064595 | Demarcation tree (*Castanea crenata*) | Village border | 1814 |
| Tsukubachoushihennsanniinkai. Tsukubachoushi-shiryoushuu-dai9hen. Ibaraki: Isebu; 1985. (in Japanese) | Kangoori | 36.192861 | 140.116757 | Line planting of pines | Divided forest | 1852 |
| Tsukubachoushihennsanniinkai. Tsukubachoushi-shiryoushuu-dai9hen. Ibaraki: Isebu; 1985. (in Japanese) | Kangoori | 36.192861 | 140.116757 | Line planting of *Cryptomeria japonica* (L.f.) D.Don | Divided forest | 1852 |
| Tsukubachoushihennsanniinkai. Tsukubachoushi-shiryoushuu-dai9hen. Ibaraki: Isebu; 1985. (in Japanese) | Mimori | 36.17016 | 140.064078 | ***Ushikoro(shi);*** *Pourthiaea villosa* (Thunb.) DC. | Paddy fields | 1852 |
| Tsukubachoushihennsanniinkai. Tsukubachoushi-shiryoushuu-dai9hen. Ibaraki: Isebu; 1985. (in Japanese) | Mimori | 36.17016 | 140.064078 | ***Ushikoro(shi);*** *Pourthiaea villosa* | Divided forest | 1852 |
| Kasumigauramachi-kyouikuiinkai. Kasumigauramachi-shuuhen-no-komonnjyo-wo-yomu. Gyousei; 1999. (in Japanese) | Sugenoya_Fukaya | 36.101812 | 140.279812 | Pine | Divided forest | 1857 |
| Tsukubachoushihennsanniinkai. Tsukubachoushi-shiryoushuu-dai9hen. Ibaraki: Isebu; 1985. (in Japanese) | Usui | 36.201342 | 140.111305 | Large *Salix* | Shrine | 1866 |
| Ushikushi. Ushikushi-shiryou-kinseiII-muratoseikatsu. Tokyo: Seikousha; 1997. (in Japanese) | Shinchi | 35.961677 | 140.120402 | Demarcation trees | Upland fields | 1867 |
| Hitachishishi-hennsanniinkai. Shinshuu-Hitachishishi-jyoukan. Tokyo: Seikousha; 1994. (in Japanese) | Ookubo_Suwa | 36.568671 | 140.607162 | Pine | Communal land | c.a. 1716-1735 |
| Ushikushishi-hennsanniinkaiminzokubukai. Ushikushishi-minzokuchousa-houkokusho3 Shimonne-Kashiwada-Higashimamiana-no-minzoku. Ibaraki: Yatabe-Insatsu; 1996. (in Japanese) | Higashimamiana | 36.018286 | 140.147913 | *Pourthiaea villosa* (the upper branches were tied into a circle as a mark) | Divided forest | na |
| Iwamamachi-kyouikuiinkai. Iwama-choushi. Ibaraki: Iwamamachi; 2002. (in Japanese) | Kamioshinobe_Shimooshinobe | 36.287583 | 140.30988 | Line planting of pines | Village border | na |
| Hitachishishi-hennsanniinkai. Shinshuu-Hitachishishi-jyoukan. Tokyo: Seikousha; 1994. (in Japanese) | na | na | na | Demarcation trees | Communal land | na |
| Hitachishishi-hennsanniinkai. Shinshuu-Hitachishishi-jyoukan. Tokyo: Seikousha; 1994. (in Japanese) | na | na | na | Burying charcoal | Communal land | na |
| Tsuchiurashishi-hennsanniinkai. Tsuchiurashishi-hennshuushiryou-dai12hen. Ibaraki: Inamoto-Insatsusho; 1970. (in Japanese) | Migimomi_Karasuyama_Arakawa_Jitsukoku | 36.006527 | 140.182211 | Valley |  | 1687 |
| Tsuchiurashishi-hennsanniinkai. Tsuchiurashishi-hennshuushiryou-dai12hen. Ibaraki: Inamoto-Insatsusho; 1970. (in Japanese) | Migimomi_Karasuyama_Arakawa_Jitsukoku | 36.006527 | 140.182211 | Well (?) |  | 1687 |
| Ishioka-shishi-hensan-iinkai. Ishioka-shishi-gekan. Tokyo: Daiichihouki; 1985. (in Japanese) | Fuchu_Murakami_Someya | 36.200198 | 140.271943 | Unspecified (signboard?) |  | 1713 |
| Ami-machi. Ami-choushi. Tokyo: Gyousei; 1983. (in Japanese) | Ami_Wakaguri | 36.02294 | 140.20951 | Bridge |  | 1611 |
| Ami-machi. Ami-choushi. Tokyo: Gyousei; 1983. (in Japanese) | Ami_Wakaguri | 36.02294 | 140.20951 | Mound |  | 1611 |
| Ami-machi. Ami-choushi. Tokyo: Gyousei; 1983. (in Japanese) | Ami_Wakaguri | 36.02294 | 140.20951 | Valley uppermost |  | 1611 |
| Ami-machi. Ami-choushi. Tokyo: Gyousei; 1983. (in Japanese) | Ami_Takaku_Oomuro_Ooiwata | 36.034557 | 140.223319 | Mound |  | 1685 |
| Ami-machi. Ami-choushi. Tokyo: Gyousei; 1983. (in Japanese) | Ami_Takaku_Oomuro_Ooiwata | 36.034557 | 140.223319 | Trench |  | 1685 |
| Ami-machi. Ami-choushi. Tokyo: Gyousei; 1983. (in Japanese) | Ami_Takaku_Oomuro_Ooiwata | 36.034557 | 140.223319 | Valley uppermost |  | 1685 |
| Ami-machi. Ami-choushi. Tokyo: Gyousei; 1983. (in Japanese) | Ami_Takaku_Oomuro_Ooiwata | 36.034557 | 140.223319 | Road |  | 1685 |
| Tsuchiurashishi-hennsanniinkai. Tsuchiurashishi-hennshuushiryou-dai12hen. Ibaraki: Inamoto-Insatsusho; 1970. (in Japanese) | Migimomi_Karasuyama_Arakawa_Jitsukoku | 36.006527 | 140.182211 | Valley uppermost |  | 1689 |
| Tsuchiurashishi-hennsanniinkai. Tsuchiurashishi-hennshuushiryou-dai12hen. Ibaraki: Inamoto-Insatsusho; 1970. (in Japanese) | Migimomi_Karasuyama_Arakawa_Jitsukoku | 36.006527 | 140.182211 | Road |  | 1689 |
| Tsuchiurashishi-hennsanniinkai. Tsuchiurashishi-hennshuushiryou-dai12hen. Ibaraki: Inamoto-Insatsusho; 1970. (in Japanese) | Migimomi_Karasuyama_Arakawa_Jitsukoku | 36.006527 | 140.182211 | Bank |  | 1689 |
| Tsuchiurashishi-hennsanniinkai. Tsuchiurashishi-hennshuushiryou-dai12hen. Ibaraki: Inamoto-Insatsusho; 1970. (in Japanese) | Arakawa_Jitsukoku_Ami_Nakamura_Karasuyama_Migimomi | 36.006527 | 140.182211 | Trench |  | 1689 |
| Ami-machi. Ami-choushi. Tokyo: Gyousei; 1983. (in Japanese) | Jyojyo_Hanawa_Iigura | 36.003719 | 140.24341 | Mound |  | 1638 |
| Ami-machi. Ami-choushi. Tokyo: Gyousei; 1983. (in Japanese) | Oppara_Kakeuma_Hanawa | 36.010608 | 140.250565 | Mound |  | 1697 |
| Ishioka-shishi-hensan-iinkai. Ishioka-shishi-gekan. Tokyo: Daiichihouki; 1985. (in Japanese) | Fuchu_Murakami_Someya | 36.200198 | 140.271943 | Signboard? |  | 1664 |
| Ishioka-shishi-hensan-iinkai. Ishioka-shishi-gekan. Tokyo: Daiichihouki; 1985. (in Japanese) | Fuchu_Murakami_Someya | 36.200198 | 140.271943 | Mound |  | 1664 |
| Ishioka-shishi-hensan-iinkai. Ishioka-shishi-gekan. Tokyo: Daiichihouki; 1985. (in Japanese) | Fuchu_Yamazaki | 36.200198 | 140.271943 | Trench |  | 1648 |
| Yasato-choushi-hensan-iinkai. Yasato-choushi. Ibaraki: Elite Insatsu; 2005. (in Japanese) | Shimobayashi_Katano_Kanasashi_Negoya | 36.226104 | 140.226164 | Old road |  | 1693 |
| Yasato-choushi-hensan-iinkai. Yasato-choushi. Ibaraki: Elite Insatsu; 2005. (in Japanese) | Uwaso_Koya_Kakioka | 36.257564 | 140.1627 | Paddy field edge |  | 1692 |
| Yasato-choushi-hensan-iinkai. Yasato-choushi. Ibaraki: Elite Insatsu; 2005. (in Japanese) | Uwaso_Koya_Kakioka | 36.257564 | 140.1627 | Stream |  | 1692 |
| Yasato-choushi-hensan-iinkai. Yasato-choushi. Ibaraki: Elite Insatsu; 2005. (in Japanese) | Uwaso_Koya_Kakioka | 36.257564 | 140.1627 | The corner where three villages meet |  | 1692 |
| Yasato-choushi-hensan-iinkai. Yasato-choushi. Ibaraki: Elite Insatsu; 2005. (in Japanese) | Kawamata_Handa | 36.20687 | 140.200629 | Mound |  | 1653 |
| Yasato-choushi-hensan-iinkai. Yasato-choushi. Ibaraki: Elite Insatsu; 2005. (in Japanese) | Kawamata_Handa | 36.20687 | 140.200629 | Mound |  | 1695 |
| Yasato-choushi-hensan-iinkai. Yasato-choushi. Ibaraki: Elite Insatsu; 2005. (in Japanese) | Kawamata_Handa | 36.20687 | 140.200629 | Takinumatatehaki? |  | 1695 |
| Yasato-choushi-hensan-iinkai. Yasato-choushi. Ibaraki: Elite Insatsu; 2005. (in Japanese) | Kawamata_Handa | 36.20687 | 140.200629 | Abandoned farmland edge |  | 1695 |
| Yasato-choushi-hensan-iinkai. Yasato-choushi. Ibaraki: Elite Insatsu; 2005. (in Japanese) | Uwaso_Kakioka | 36.249673 | 140.164738 | Mound |  | 1692 |
| Yasato-choushi-hensan-iinkai. Yasato-choushi. Ibaraki: Elite Insatsu; 2005. (in Japanese) | Shibauchi_Katano_Negoya | 36.17996 | 140.17099 | Road |  | 1688 |
| Yasato-choushi-hensan-iinkai. Yasato-choushi. Ibaraki: Elite Insatsu; 2005. (in Japanese) | Shimoaoyagi_Tsukioka | 36.206986 | 140.170416 | Ridge? |  | 1650 |
| Yasato-choushi-hensan-iinkai. Yasato-choushi. Ibaraki: Elite Insatsu; 2005. (in Japanese) | Shimoaoyagi_Tsukioka | 36.206986 | 140.170416 | *Kuwagata-wotsuke*? |  | 1650 |
| Yasato-choushi-hensan-iinkai. Yasato-choushi. Ibaraki: Elite Insatsu; 2005. (in Japanese) | Shimoaoyagi_Tsukioka | 36.206986 | 140.170416 | Large road |  | 1650 |
| Ibaraki-choushi-hensan-iinkai. Ibaraki-choushi-tsuushihen. Tokyo: Seikousha; 1995 (in Japanese) | Nagatoro_Kashiwai_Nikoda_Kawamata＿Kurisaki_Noso | 36.305723 | 140.357043 | *Sumizuka* (mound filled with charcoal?) |  | 1666 |
| Ogawa-choushi-hensan-iinkai. Ogawa-choushi-jyoukan. Tokyo: Daiichihouki; 1982. (in Japanese) | Tachinobe_Miyata | 36.17706 | 140.35587 | Road |  | 1668 |
| Ogawa-choushi-hensan-iinkai. Ogawa-choushi-jyoukan. Tokyo: Daiichihouki; 1982. (in Japanese) | Tachinobe_Miyata | 36.17706 | 140.35587 | Trench |  | 1668 |
| Ogawa-choushi-hensan-iinkai. Ogawa-choushi-jyoukan. Tokyo: Daiichihouki; 1982. (in Japanese) | Tachinobe_Miyata | 36.17706 | 140.35587 | Yatsukiri (valley?) |  | 1668 |
| Iwamamachi-kyouikuiinkai. Iwama-choushi-shiryoushu-komonjyo-daiisshu. Ibaraki: Iwamamachi-kyouikuiinkai; 1992. (in Japanese) | Kamioshinobe_Shimooshinobe | 36.287336 | 140.307504 | *Sakaiyaki* (road burning) |  | 1848 |
| Iwamamachi-kyouikuiinkai. Iwama-choushi. Ibaraki: Iwamamachi; 2002. (in Japanese) | Oogoyama_Haji | 36.312079 | 140.301384 | Mound |  | 1656 |
| Iwamamachi-kyouikuiinkai. Iwama-choushi. Ibaraki: Iwamamachi; 2002. (in Japanese) | Iwama_Oogoyama_Haji | 36.30009 | 140.29896 | Mound |  | 1656 |
| Iwamamachi-kyouikuiinkai. Iwama-choushi. Ibaraki: Iwamamachi; 2002. (in Japanese) | Haji_Oshinobe | 36.301623 | 140.30164 | Mound |  | c.a. 1720 |
| Iwamamachi-kyouikuiinkai. Iwama-choushi. Ibaraki: Iwamamachi; 2002. (in Japanese) | Kamioshinobe_Shimooshinobe | 36.287583 | 140.30988 | Mound |  | na |
| Iwamamachi-kyouikuiinkai. Iwama-choushi. Ibaraki: Iwamamachi; 2002. (in Japanese) | Izumi_Maie | 36.277176 | 140.273868 | Stream |  | 1637 |
| Kasumigauramachi-kyouikuiinkai. Kasumigauramachi-shuuhen-no-komonnjyo-wo-yomu. Gyousei; 1999. (in Japanese) | Sugenoya_Fukaya | 36.101812 | 140.279812 | Road |  | 1857 |
| Chiyodamura-kyouikuiinkai. Chiyoda-sonshi. Ibaraki: Kikuchi Insatsu; 1970. (in Japanese) | Yukiiri_Nagai_Hongou | 36.16491 | 140.187651 | Mound for the Koshin ceremony |  | 1653 |
| Kamisumachi. Kamisu-choushi-jyoukan. Tokyo: Seikousha; 1988. (in Japanese) | Ikisu_Takahama_Ishigami_Tabata_Kisaki_Mizoguchi_Igiri_Shimohataki_Hiraizumi_Ga_Tsutsui_Nagasu_Izumigawa | 35.893007 | 140.643144 | Mound |  | 1844 |
| Kitaibaraki-shishi-hensaniinkai. Kitaibaraki-shishi-jyoukan. Tokyo: Seikousha; 1988. (in Japanese) | Kubota_Sekimotokamino_Fukuda_Hattan | 36.867633 | 140.761172 | Ridge |  | c.a. 1710 |
| Kitaibaraki-shishi-hensaniinkai. Kitaibaraki-shishi-jyoukan. Tokyo: Seikousha; 1988. (in Japanese) | Kubota_Sekimotokamino_Fukuda_Hattan | 36.867633 | 140.761172 | Stream |  | c.a. 1710 |
| Kitaibaraki-shishi-hensaniinkai. Kitaibaraki-shishi-jyoukan. Tokyo: Seikousha; 1988. (in Japanese) | Kubota_Sekimotokamino_Fukuda_Hattan | 36.867633 | 140.761172 | Levee |  | c.a. 1710 |
| Sanwa-choushi-hensaniinkai. Sanwachoushi-shiryouhen-kinsei. Tokyo: Seikousha; 1992. (in Japanese) | Yagai_Higashiyamata | 36.149948 | 139.816841 | Mound |  | 1661 |
| Sanwa-choushi-hensaniinkai. Sanwachoushi-shiryouhen-kinsei. Tokyo: Seikousha; 1992. (in Japanese) | Yagai_Higashiyamata | 36.149948 | 139.816841 | Road |  | 1661 |
| Sanwa-choushi-hensaniinkai. Sanwachoushi-shiryouhen-kinsei. Tokyo: Seikousha; 1992. (in Japanese) | Oowada_Kamioono | 36.198054 | 139.788215 | Trench |  | 1714 |
| Sanwa-choushi-hensaniinkai. Sanwachoushi-shiryouhen-kinsei. Tokyo: Seikousha; 1992. (in Japanese) | Kamiwada | 36.226749 | 139.816368 | Trench |  | 1741 |
| Souwa-choushi-hensaniinkai. Souwa-choushi-shiryouhen-kinsei. Ibaraki: Higashinihoninsatsu. | Oowada_Kamioono_Inamiya_Kamikatata_Shimokatata | 36.218462 | 139.803187 | Trench |  | 1673 |
| Goka-choushi-hensaniinkai. Choushi-Goka-no-seikatsushi shiryou1. Tokyo: Gyousei; 2011. (in Japanese) | Motokurihashi_Shinkouya | 36.114046 | 139.739754 | Earth tower |  | 1670 |
| Goka-choushi-hensaniinkai. Choushi-Goka-no-seikatsushi shiryou1. Tokyo: Gyousei; 2011. (in Japanese) | Motokurihashi_Shinkouya | 36.114046 | 139.739754 | Bank |  | 1670 |
| Goka-choushi-hensaniinkai. Choushi-Goka-no-seikatsushi shiryou1. Tokyo: Gyousei; 2011. (in Japanese) | Motokurihashi_Shinkouya | 36.114046 | 139.739754 | Road |  | 1670 |
| Goka-choushi-hensaniinkai. Choushi-Goka-no-seikatsushi shiryou1. Tokyo: Gyousei; 2011. (in Japanese) | Kawatsuma_Motokurihashi | 36.122159 | 139.727275 | Embankment |  | 1753 |
| Goka-choushi-hensaniinkai. Choushi-Goka-no-seikatsushi shiryou1. Tokyo: Gyousei; 2011. (in Japanese) | Kawatsuma_Motokurihashi | 36.122159 | 139.727275 | Road |  | 1753 |
| Sakai-choushi-hensaniinkai. Shimousasakai-no-seikatsushi shiryouhen kinseiII mura-no-seikatsu. Maebashi: Asahiinsatsu; 2002. (in Japanese) | Modo_Kaneoka_Nittado | 36.072696 | 139.809634 | Trench |  | 1717 |
| Ushikushi. Ushiku-shishi-kinsei. Mito: Akebono-insatsusha; 2002. (in Japanese) | Kashiwada_Ushiku_Jyouchuu_Tooyama | 36..980199 | 140.153476 | Mound |  | 1656 |
| Ushikushi. Ushiku-shishi-kinsei. Mito: Akebono-insatsusha; 2002. (in Japanese) | Kuno_Kosaka_Shoujiki_Shimada | 35.96931 | 140.20123 | Mound |  | 1670 |
| Ushikushi. Ushiku-shishi-kinsei. Mito: Akebono-insatsusha; 2002. (in Japanese) | Fukuda_Kosaka | 35.969926 | 140.200917 | Bank |  | 1712 |
| Ushikushi. Ushikushi-shiryou-kinseiII-muratoseikatsu. Tokyo: Seikousha; 1997. (in Japanese) | Kuno_Kosaka_Shoujiki_Shimada | 35.959603 | 140.226418 | Mound |  | 1740 |
| Ushikushi. Ushikushi-shiryou-kinseiII-muratoseikatsu. Tokyo: Seikousha; 1997. (in Japanese) | Kuno_Kosaka_Shoujiki_Shimada | 35.959603 | 140.226418 | Trench |  | 1740 |
| Kukizaki-choushi-hennsanniinkai. Kukizaki-choushi-hennsanniinkai-shiryou-kinsei-kindaishi-shiryoushu. Ibaraki: Isebu; 1993. (in Japanese) | Takasaki_Oguki_Taguu_Amabougi | 35.982564 | 140.132076 | Mound |  | 1656 |
| Ushikushi. Ushikushi-shiryou-kinseiII-muratoseikatsu. Tokyo: Seikousha; 1997. (in Japanese) | Kawarashiro_Kashiwada | 35.914396 | 140.149458 | Trench |  | 1680 |
| Ushikushi. Ushikushi-shiryou-kinseiII-muratoseikatsu. Tokyo: Seikousha; 1997. (in Japanese) | Kawarashiro_Kashiwada | 35.914396 | 140.149458 | Mound |  | 1680 |
| Ushikushi. Ushikushi-shiryou-kinseiII-muratoseikatsu. Tokyo: Seikousha; 1997. (in Japanese) | Kawarashiro_Kashiwada | 35.914396 | 140.149458 | Road |  | 1680 |
| Ushikushi. Ushikushi-shiryou-kinseiII-muratoseikatsu. Tokyo: Seikousha; 1997. (in Japanese) | Kawarashiro_Kashiwada | 35.914396 | 140.149458 | Unspecified way in upland fields |  | 1680 |
| Ushikushi. Ushikushi-shiryou-kinseiII-muratoseikatsu. Tokyo: Seikousha; 1997. (in Japanese) | Oowada_Nakane_Shimone | 36.001222 | 140.158682 | Paddy field |  | 1687 |
| Ushikushi. Ushikushi-shiryou-kinseiII-muratoseikatsu. Tokyo: Seikousha; 1997. (in Japanese) | Oowada_Nakane_Shimone | 36.001222 | 140.158682 | Stream? |  | 1687 |
| Ushikushi. Ushikushi-shiryou-kinseiII-muratoseikatsu. Tokyo: Seikousha; 1997. (in Japanese) | Oowada_Nakane_Shimone | 36.001222 | 140.158682 | Road |  | 1687 |
| Ushikushi. Ushikushi-shiryou-kinseiII-muratoseikatsu. Tokyo: Seikousha; 1997. (in Japanese) | Kosaka_Okami | 35.977169 | 140.188078 | Mound |  | 1718 |
| Ushikushi. Ushikushi-shiryou-kinseiII-muratoseikatsu. Tokyo: Seikousha; 1997. (in Japanese) | Kosaka_Okami | 35.977169 | 140.188078 | Paddy edge |  | 1718 |
| Ushikushi. Ushikushi-shiryou-kinseiII-muratoseikatsu. Tokyo: Seikousha; 1997. (in Japanese) | Kosaka_Okami | 35.977169 | 140.188078 | Road intersection |  | 1718 |
| Ushikushi. Ushikushi-shiryou-kinseiII-muratoseikatsu. Tokyo: Seikousha; 1997. (in Japanese) | Bessho_Nareuma | 35.926133 | 140.178497 | Mound |  | 1820 |
| Iwase-choushi-hennsanniinkai. Iwase-choushi-shiryouhen. Iwase: Ishizakiinsatsu; 1983. (in Japanese) | Kadoke_Tomiya | 36.379618 | 140.093633 | Ridge |  | 1670 |
| Iwase-choushi-hennsanniinkai. Iwase-choushi-shiryouhen. Iwase: Ishizakiinsatsu; 1983. (in Japanese) | Kadoke_Tomiya | 36.379618 | 140.093633 | Ditch |  | 1670 |
| Iwase-choushi-hennsanniinkai. Iwase-choushi-shiryouhen. Iwase: Ishizakiinsatsu; 1983. (in Japanese) | Kadoke_Tomiya | 36.379618 | 140.093633 | Mountain foot |  | 1670 |
| Iwase-choushi-hennsanniinkai. Iwase-choushi-shiryouhen. Iwase: Ishizakiinsatsu; 1983. (in Japanese) | Irino_Kadoke | 36.394289 | 140.120945 | Edge of woods |  | 1670 |
| Iwase-choushi-hennsanniinkai. Iwase-choushi-shiryouhen. Iwase: Ishizakiinsatsu; 1983. (in Japanese) | Irino_Kadoke | 36.394289 | 140.120945 | Stream |  | 1670 |
| Iwase-choushi-hennsanniinkai. Iwase-choushi-shiryouhen. Iwase: Ishizakiinsatsu; 1983. (in Japanese) | Irino_Kadoke | 36.394289 | 140.120945 | Road |  | 1670 |
| Iwase-choushi-hennsanniinkai. Iwase-choushi-shiryouhen. Iwase: Ishizakiinsatsu; 1983. (in Japanese) | Irino_Kadoke | 36.394289 | 140.120945 | Ridge |  | 1670 |
| Iwase-choushi-hennsanniinkai. Iwase-choushi-shiryouhen. Iwase: Ishizakiinsatsu; 1983. (in Japanese) | Kadoke_Yamamoto | 36.411282 | 140.117786 | Mound |  | 1685 |
| Iwase-choushi-hennsanniinkai. Iwase-choushi-shiryouhen. Iwase: Ishizakiinsatsu; 1983. (in Japanese) | Kadoke_Yamamoto | 36.411282 | 140.117786 | Ridge |  | 1685 |
| Iwase-choushi-hennsanniinkai. Iwase-choushi-shiryouhen. Iwase: Ishizakiinsatsu; 1983. (in Japanese) | Kadoke_Yamamoto | 36.411282 | 140.117786 | Jizo statue |  | 1685 |
| Iwase-choushi-hennsanniinkai. Iwase-choushi-shiryouhen. Iwase: Ishizakiinsatsu; 1983. (in Japanese) | Tomiya_Yamamoto | 36.407438 | 140.109363 | Mound |  | 1671 |
| Iwase-choushi-hennsanniinkai. Iwase-choushi-shiryouhen. Iwase: Ishizakiinsatsu; 1983. (in Japanese) | Tomiya_Yamamoto | 36.407438 | 140.109363 | Three-way junction (ridge?) |  | 1671 |
| Iwase-choushi-hennsanniinkai. Iwase-choushi-shiryouhen. Iwase: Ishizakiinsatsu; 1983. (in Japanese) | Tomiya_Yamamoto | 36.407438 | 140.109363 | Stream |  | 1671 |
| Iwase-choushi-hennsanniinkai. Iwase-choushi-shiryouhen. Iwase: Ishizakiinsatsu; 1983. (in Japanese) | Tomiya_Yamamoto | 36.407438 | 140.109363 | Peak |  | 1671 |
| Iwase-choushi-hennsanniinkai. Iwase-choushi-shiryouhen. Iwase: Ishizakiinsatsu; 1983. (in Japanese) | Yomogita_Hongou | 36.362427 | 140.044661 | Mound |  | 1695 |
| Iwase-choushi-hennsanniinkai. Iwase-choushi-shiryouhen. Iwase: Ishizakiinsatsu; 1983. (in Japanese) | Fukuzaki_Kameoka_Manaka_Iida | 36.383667 | 140.137098 | Road |  | 1702 |
| Iwase-choushi-hennsanniinkai. Iwase-choushi-shiryouhen. Iwase: Ishizakiinsatsu; 1983. (in Japanese) | Fukuzaki_Kameoka_Manaka_Iida | 36.383667 | 140.137098 | Paddy field and upland field |  | 1702 |
| Iwase-choushi-hennsanniinkai. Iwase-choushi-shiryouhen. Iwase: Ishizakiinsatsu; 1983. (in Japanese) | Fukuzaki_Kameoka_Manaka_Iida | 36.383667 | 140.137098 | Peak |  | 1702 |
| Iwase-choushi-hennsanniinkai. Iwase-choushi-shiryouhen. Iwase: Ishizakiinsatsu; 1983. (in Japanese) | Fukuzaki_Kameoka_Manaka_Iida | 36.383667 | 140.137098 | Mound |  | 1702 |
| Iwase-choushi-hennsanniinkai. Iwase-choushi-shiryouhen. Iwase: Ishizakiinsatsu; 1983. (in Japanese) | Ooizumi | 36.386246 | 140.076613 | Road |  | 1709 |
| Iwase-choushi-hennsanniinkai. Iwase-choushi-shiryouhen. Iwase: Ishizakiinsatsu; 1983. (in Japanese) | Shimoizumi_Tsutsuminoue | 36.366617 | 140.058581 | Mound |  | 1748 |
| Iwase-choushi-hennsanniinkai. Iwase-choushi-shiryouhen. Iwase: Ishizakiinsatsu; 1983. (in Japanese) | Motogi_Inuta | 36.340819 | 140.102049 | Trench |  | 1871 |
| Iwase-choushi-hennsanniinkai. Iwase-choushi-shiryouhen. Iwase: Ishizakiinsatsu; 1983. (in Japanese) | Motogi_Inuta | 36.340819 | 140.102049 | Mound |  | 1871 |
| Iwase-choushi-hennsanniinkai. Iwase-choushi-shiryouhen. Iwase: Ishizakiinsatsu; 1983. (in Japanese) | Motogi_Inuta | 36.340819 | 140.102049 | Embankment |  | 1871 |
| Iwase-choushi-hennsanniinkai. Iwase-choushi-shiryouhen. Iwase: Ishizakiinsatsu; 1983. (in Japanese) | Motogi_Inuta | 36.340819 | 140.102049 | Stream |  | 1871 |
| Iwase-choushi-hennsanniinkai. Iwase-choushi-shiryouhen. Iwase: Ishizakiinsatsu; 1983. (in Japanese) | Motogi_Inuta | 36.340819 | 140.102049 | Peak |  | 1871 |
| Iijima M. Yamato-sonshi-yokou. Yamato-mura: Ishizakiinsatsu; 1996. (in Japanese) | Aoki_Inuta | 36.35725 | 140.09238 | Mound |  | 1848 |
| Iijima M. Yamato-sonshi-yokou. Yamato-mura: Ishizakiinsatsu; 1996. (in Japanese) | Aoki_Inuta | 36.35725 | 140.09238 | Pile |  | 1848 |
| Iijima M. Yamato-sonshi-yokou. Yamato-mura: Ishizakiinsatsu; 1996. (in Japanese) | Aoki_Inuta | 36.35725 | 140.09238 | Rock |  | 1848 |
| Iijima M. Yamato-sonshi-yokou. Yamato-mura: Ishizakiinsatsu; 1996. (in Japanese) | Aoki_Inuta | 36.35725 | 140.09238 | Stone hut |  | 1848 |
| Iijima M. Yamato-sonshi-yokou. Yamato-mura: Ishizakiinsatsu; 1996. (in Japanese) | Haneta_Aoki | 36.339456 | 140.09201 | Mound |  | 1854 |
| Nanakai-sonshi-hennsanniinkai. Nanakaimura-no-rekishi. Tokyo: Seikousha; 2005. (in Japanese) | Shiogo_Akasawa | 36.521858 | 140.287816 | Peak |  | 1683 |
| Nanakai-sonshi-hennsanniinkai. Nanakaimura-no-rekishi. Tokyo: Seikousha; 2005. (in Japanese) | Shiogo_Akasawa | 36.521858 | 140.287816 | Ridge |  | 1683 |
| Nanakai-sonshi-hennsanniinkai. Nanakaimura-no-rekishi. Tokyo: Seikousha; 2005. (in Japanese) | Shiogo_Akasawa | 36.521858 | 140.287816 | Pile |  | 1683 |
| Nanakai-sonshi-hennsanniinkai. Nanakaimura-no-rekishi. Tokyo: Seikousha; 2005. (in Japanese) | Shiogo_Ogachi_Tokura | 36.463044 | 140.265948 | Mound |  | 1621 |
| Nanakai-sonshi-hennsanniinkai. Nanakaimura-no-rekishi. Tokyo: Seikousha; 2005. (in Japanese) | Akasawa_Tokura | 36.465964 | 140.231424 | Mound |  | 1686 |
| Akeno-choushi-hennsanniinkai. Akeno-choushi-shiryou dai21shuu kinseishiryou6 Akeno-no-soshoudeiri1. Fukuoka: Saneiinsatsu; 1994. (in Japanese) | Ebigashima_Kuwayama | 36.29332 | 140.0324 | Mound |  | 1649 |
| Akeno-choushi-hennsanniinkai. Akeno-choushi-shiryou dai21shuu kinseishiryou6 Akeno-no-soshoudeiri1. Fukuoka: Saneiinsatsu; 1994. (in Japanese) | Miyayama_Miyago | 36.25841 | 140.05148 | Road |  | 1693 |
| Akeno-choushi-hennsanniinkai. Akeno-choushi-shiryou dai21shuu kinseishiryou6 Akeno-no-soshoudeiri1. Fukuoka: Saneiinsatsu; 1994. (in Japanese) | Kuramochi_Teraueno | 36.235362 | 140.02982 | Road |  | 1702 |
| Akeno-choushi-hennsanniinkai. Akeno-choushi-shiryou dai21shuu kinseishiryou6 Akeno-no-soshoudeiri1. Fukuoka: Saneiinsatsu; 1994. (in Japanese) | Kuramochi_Teraueno | 36.235362 | 140.02982 | Trench |  | 1702 |
| Akeno-choushi-hennsanniinkai. Akeno-choushi-shiryou dai21shuu kinseishiryou6 Akeno-no-soshoudeiri1. Fukuoka: Saneiinsatsu; 1994. (in Japanese) | Kuramochi_Teraueno_Nakaueno_Mukouueno_Narui_Ebigashima_Matsubara_Nakane_Sanoudou_Tajuku_Nekoshima_Uchiyodo_Nabeyama | 36.275266 | 140.028992 | Pond |  | 1702 |
| Akeno-choushi-hennsanniinkai. Akeno-choushi-shiryou dai21shuu kinseishiryou6 Akeno-no-soshoudeiri1. Fukuoka: Saneiinsatsu; 1994. (in Japanese) | Kuramochi_Teraueno_Nakaueno_Mukouueno_Narui_Ebigashima_Matsubara_Nakane_Sanoudou_Tajuku_Nekoshima_Uchiyodo_Nabeyama | 36.275266 | 140.028992 | Bank |  | 1702 |
| Akeno-choushi-hennsanniinkai. Akeno-choushi-shiryou dai21shuu kinseishiryou6 Akeno-no-soshoudeiri1. Fukuoka: Saneiinsatsu; 1994. (in Japanese) | Shimokawanago | 36.272461 | 139.993093 | Trench |  | 1713 |
| Akeno-choushi-hennsanniinkai. Akeno-choushi-shiryou dai21shuu kinseishiryou6 Akeno-no-soshoudeiri1. Fukuoka: Saneiinsatsu; 1994. (in Japanese) | Ebie_Higashihozue_ | 36.243671 | 139.998535 | Trench (unclear whether it is a border or not) |  | 1713 |
| Akeno-choushi-hennsanniinkai. Akeno-choushi-shiryou dai21shuu kinseishiryou6 Akeno-no-soshoudeiri1. Fukuoka: Saneiinsatsu; 1994. (in Japanese) | Ebie_Higashihozue_ | 36.243671 | 139.998535 | Pile |  | 1713 |
| Sekijyou-choushi-hennsanniinkai. Sekijyou-choushi-shiryouhen1. Tokyo: Seikousha; 1983. (in Japanese) | Takasai | 36.20465 | 139.997346 | Pile |  | 1748 |
| Sekijyou-choushi-hennsanniinkai. Sekijyou-choushi-shiryouhen1. Tokyo: Seikousha; 1983. (in Japanese) | Nakaueno | 36.207088 | 140.002224 | Mound |  | 1850 |
| Sekijyou-choushi-hennsanniinkai. Sekijyou-choushi-shiryouhen1. Tokyo: Seikousha; 1983. (in Japanese) | Nakaueno | 36.207088 | 140.002224 | Pile |  | 1850 |
| Kukizaki-choushi-hennsanniinkai. Kukizaki-choushi-hennsanniinkai-shiryou-kinsei-kindaishi-shiryoushu. Ibaraki: Isebu; 1993. (in Japanese) | Ooi_Hinosawa | 36.018082 | 140.130507 | Bank |  | 1700 |
| Kukizaki-choushi-hennsanniinkai. Kukizaki-choushi-hennsanniinkai-shiryou-kinsei-kindaishi-shiryoushu. Ibaraki: Isebu; 1993. (in Japanese) | Ooi_Hinosawa | 36.018082 | 140.130507 | Corner where two upland fields meet |  | 1700 |
| Kukizaki-choushi-hennsanniinkai. Kukizaki-choushi-hennsanniinkai-shiryou-kinsei-kindaishi-shiryoushu. Ibaraki: Isebu; 1993. (in Japanese) | Amabouki_Takasaki_Oguki_Sugama_Ushiku_Jyouchuu_Tooyama | 35.989867 | 140.125872 | Unspecified way |  | 1700 |
| Tsukubachoushihennsanniinkai. Tsukubachoushi-shiryoushuu-dai9hen. Ibaraki: Isebu; 1985. (in Japanese) | Tsukuba_Numata | 36.210891 | 140.093186 | Stream |  | 1663 |
| Tsukubachoushihennsanniinkai. Tsukubachoushi-shiryoushuu-dai9hen. Ibaraki: Isebu; 1985. (in Japanese) | Tsukuba_Numata | 36.210891 | 140.093186 | Waterfall |  | 1663 |
| Tsukubachoushihennsanniinkai. Tsukubachoushi-shiryoushuu-dai9hen. Ibaraki: Isebu; 1985. (in Japanese) | Tsukuba_Numata | 36.210891 | 140.093186 | Mountain edge |  | 1663 |
| Tsukubachoushihennsanniinkai. Tsukubachoushi-shiryoushuu-dai9hen. Ibaraki: Isebu; 1985. (in Japanese) | Usui_Kangoori | 36.204205 | 140.114418 | Ridge |  | 1706 |
| Tsukubachoushihennsanniinkai. Tsukubachoushi-shiryoushuu-dai9hen. Ibaraki: Isebu; 1985. (in Japanese) | Usui_Kangoori | 36.204205 | 140.114418 | Road |  | 1706 |
| Tsukubachoushihennsanniinkai. Tsukubachoushi-shiryoushuu-dai9hen. Ibaraki: Isebu; 1985. (in Japanese) | Usui_Kangoori | 36.204205 | 140.114418 | Edge of residence area |  | 1706 |
| Tsukubachoushihennsanniinkai. Tsukubachoushi-shiryoushuu-dai9hen. Ibaraki: Isebu; 1985. (in Japanese) | Usui_Kangoori | 36.204205 | 140.114418 | Mound |  | 1706 |
| Tsukubachoushihennsanniinkai. Tsukubachoushi-shiryoushuu-dai9hen. Ibaraki: Isebu; 1985. (in Japanese) | Usui | 36.201342 | 140.111305 | Ridge |  | 1866 |
| Tsukubachoushihennsanniinkai. Tsukubachoushi-shiryoushuu-dai9hen. Ibaraki: Isebu; 1985. (in Japanese) | Usui | 36.201342 | 140.111305 | Pile |  | 1866 |
| Tsukubachoushihennsanniinkai. Tsukubachoushi-shiryoushuu-dai9hen. Ibaraki: Isebu; 1985. (in Japanese) | Houjyou_Hirasawa | 36.183193 | 140.124869 | Stream |  | 1876 |
| Tsukubachoushihennsanniinkai. Tsukubachoushi-shiryoushuu-dai9hen. Ibaraki: Isebu; 1985. (in Japanese) | Houjyou_Hirasawa | 36.183193 | 140.124869 | Ridge |  | 1876 |
| Tsukubachoushihennsanniinkai. Tsukubachoushi-shiryoushuu-dai9hen. Ibaraki: Isebu; 1985. (in Japanese) | Obata_the other eight villages | 36.211230 | 140.122669 | Pile |  | 1871 |
| Tsukubachoushihennsanniinkai. Tsukubachoushi-shiryoushuu-dai9hen. Ibaraki: Isebu; 1985. (in Japanese) | Kowada_Oda | 36.159862 | 140.112777 | Unspecified way |  | 1631 |
| Tsukubachoushihennsanniinkai. Tsukubachoushi-shiryoushuu-dai9hen. Ibaraki: Isebu; 1985. (in Japanese) | Oda_Oota | 36.158272 | 140.113953 | Mound |  | 1690 |
| Tsukubachoushihennsanniinkai. Tsukubachoushi-shiryoushuu-dai9hen. Ibaraki: Isebu; 1985. (in Japanese) | Oda_Oota | 36.158272 | 140.113953 | Ridge? |  | 1690 |
| Tsukubachoushihennsanniinkai. Tsukubachoushi-shiryoushuu-dai9hen. Ibaraki: Isebu; 1985. (in Japanese) | Yamaguchi_Kowada | 36.167121 | 140.118152 | Road |  | 1867 |
| Tsukubachoushihennsanniinkai. Tsukubachoushi-shiryoushuu-dai9hen. Ibaraki: Isebu; 1985. (in Japanese) | Yamaguchi_Kowada | 36.167121 | 140.118152 | Stream |  | 1867 |
| Tsukubachoushihennsanniinkai. Tsukubachoushi-shiryoushuu-dai9hen. Ibaraki: Isebu; 1985. (in Japanese) | Yamaguchi_Kowada | 36.167121 | 140.118152 | Ridge |  | 1867 |
| Tsukubachoushihennsanniinkai. Tsukubachoushi-shiryoushuu-dai9hen. Ibaraki: Isebu; 1985. (in Japanese) | Kamisugama_Mukouueno | 36.206904 | 140.048583 | Edge of upland field |  | 1719 |
| Tsukubachoushihennsanniinkai. Tsukubachoushi-shiryoushuu-dai9hen. Ibaraki: Isebu; 1985. (in Japanese) | Kamisugama_Mukouueno | 36.206904 | 140.048583 | Mound |  | 1719 |
| Tsukubachoushihennsanniinkai. Tsukubachoushi-shiryoushuu-dai9hen. Ibaraki: Isebu; 1985. (in Japanese) | Kamisugama_Mukouueno | 36.206904 | 140.048583 | Road |  | 1719 |
| Tsukubachoushihennsanniinkai. Tsukubachoushi-shiryoushuu-dai9hen. Ibaraki: Isebu; 1985. (in Japanese) | Tsukuriya | 36.180923 | 140.044718 | Mound |  | 1846 |
| Tsukubachoushihennsanniinkai. Tsukubachoushi-shiryoushuu-dai9hen. Ibaraki: Isebu; 1985. (in Japanese) | Tsukuriya | 36.180923 | 140.044718 | Unspecified (signboard?) |  | 1846 |
| Tsukubachoushihennsanniinkai. Tsukubachoushi-shiryoushuu-dai9hen. Ibaraki: Isebu; 1985. (in Japanese) | Tsukuriya | 36.180923 | 140.044718 | Pile |  | 1846 |
| Tsukubachoushihennsanniinkai. Tsukubachoushi-shiryoushuu-dai9hen. Ibaraki: Isebu; 1985. (in Japanese) | Maeno_Mimori | 36.15533 | 140.060336 | Unspecified (signboard?) |  | 1690 |
| Tsukubachoushihennsanniinkai. Tsukubachoushi-shiryoushuu-dai9hen. Ibaraki: Isebu; 1985. (in Japanese) | Maeno_Mimori | 36.15533 | 140.060336 | Edge of woods |  | 1694 |
| Tsukubachoushihennsanniinkai. Tsukubachoushi-shiryoushuu-dai9hen. Ibaraki: Isebu; 1985. (in Japanese) | Maeno_Mimori | 36.15533 | 140.060336 | Mound |  | 1694 |
| Tsukubachoushihennsanniinkai. Tsukubachoushi-shiryoushuu-dai9hen. Ibaraki: Isebu; 1985. (in Japanese) | Maeno_Mimori | 36.15533 | 140.060336 | Road |  | 1694 |
| Tsukubachoushihennsanniinkai. Tsukubachoushi-shiryoushuu-dai9hen. Ibaraki: Isebu; 1985. (in Japanese) | Maeno_Mimori | 36.15533 | 140.060336 | Unspecified (signboard?) |  | 1694 |
| Tsukubachoushihennsanniinkai. Tsukubachoushi-shiryoushuu-dai9hen. Ibaraki: Isebu; 1985. (in Japanese) | Mimori | 36.17016 | 140.064078 | Mound |  | 1852 |
| Tsuchiurashi-kyouikuiinkai. Tsuchiurashi-bikou-dai5kan. Ibaraki: Tsuchiurashi-kyouikuiinkai; 1996. (in Japanese) | Sakemaru_Omonoi_Karima_Hiratsuka | 36.082572 | 140.08232 | Bulletin board |  | 1688 |
| Tsuchiurashi-kyouikuiinkai. Tsuchiurashi-bikou-dai5kan. Ibaraki: Tsuchiurashi-kyouikuiinkai; 1996. (in Japanese) | Sakemaru_Omonoi_Karima_Hiratsuka | 36.082572 | 140.08232 | Edge of woods |  | 1688 |
| Tsuchiurashi-kyouikuiinkai. Tsuchiurashi-bikou-dai5kan. Ibaraki: Tsuchiurashi-kyouikuiinkai; 1996. (in Japanese) | Kurakake_Hanamuro_Onozaki | 36.082663 | 140.128445 | Ditch |  | 1688 |
| Tsuchiurashi-kyouikuiinkai. Tsuchiurashi-bikou-dai5kan. Ibaraki: Tsuchiurashi-kyouikuiinkai; 1996. (in Japanese) | Kurakake_Hanamuro_Onozaki | 36.082663 | 140.128445 | Road |  | 1688 |
| Tsuchiurashi-kyouikuiinkai. Tsuchiurashi-bikou-dai5kan. Ibaraki: Tsuchiurashi-kyouikuiinkai; 1996. (in Japanese) | Kurakake_Hanamuro_Onozaki | 36.082663 | 140.128445 | Edge of upland field |  | 1688 |
| Tsuchiurashi-kyouikuiinkai. Tsuchiurashi-bikou-dai5kan. Ibaraki: Tsuchiurashi-kyouikuiinkai; 1996. (in Japanese) | Kurakake_Hanamuro_Onozaki | 36.082663 | 140.128445 | Mound |  | 1688 |
| Tsuchiurashi-kyouikuiinkai. Tsuchiurashi-bikou-dai5kan. Ibaraki: Tsuchiurashi-kyouikuiinkai; 1996. (in Japanese) | Kurakake_Hanamuro_Onozaki | 36.082663 | 140.128445 | Swamp |  | 1688 |
| Tsuchiurashi-kyouikuiinkai. Tsuchiurashi-bikou-dai5kan. Ibaraki: Tsuchiurashi-kyouikuiinkai; 1996. (in Japanese) | Kurakake_Hanamuro_Onozaki | 36.082663 | 140.128445 | Valley |  | 1688 |
| Tsuchiurashi-kyouikuiinkai. Tsuchiurashi-bikou-dai5kan. Ibaraki: Tsuchiurashi-kyouikuiinkai; 1996. (in Japanese) | Matsuduka_Iida | 36.10496 | 140.161819 | Mound |  | 1758 |
| Tsuchiurashi-kyouikuiinkai. Tsuchiurashi-bikou-dai5kan. Ibaraki: Tsuchiurashi-kyouikuiinkai; 1996. (in Japanese) | Iida_seven villages in Niihari County | 36.098735 | 140.16794 | Mound |  | 1759 |
| Tsuchiurashi-kyouikuiinkai. Tsuchiurashi-bikou-dai5kan. Ibaraki: Tsuchiurashi-kyouikuiinkai; 1996. (in Japanese) | Iida_seven villages in Niihari County | 36.098735 | 140.16794 | Mound with stone monument |  | 1759 |
| Tsuchiurashishi-hennsanniinkai. Tsuchiurashishi-hennshuushiryou-dai12hen. Ibaraki: Inamoto-Insatsusho; 1970. (in Japanese) | Arakawa_Migimomi_Karasuyama | 36.02894 | 140.17119 | Mound |  | 1685 |
| Tsuchiurashishi-hennsanniinkai. Tsuchiurashishi-hennshuushiryou-dai12hen. Ibaraki: Inamoto-Insatsusho; 1970. (in Japanese) | Arakawa_Migimomi_Karasuyama | 36.02894 | 140.17119 | Road |  | 1685 |
| Tsuchiurashishi-hennsanniinkai. Tsuchiurashishi-hennshuushiryou-dai12hen. Ibaraki: Inamoto-Insatsusho; 1970. (in Japanese) | Migimomi_Karasuyama_Arakawa_Jitsukoku | 36.006527 | 140.182211 | Mound |  | 1687 |
| Niiharisonshi-hennsannjimukyoku. Niiharisonshi-shiryoushuu-dai4hen. Ibaraki: Takahashi-Insatsu; 1985. (in Japanese) | Nagai_Hongou_Yukiiri_Kamisaya | 36.16736 | 140.17486 | Ridge |  | 1653 |
| Niiharisonshi-hennsannjimukyoku. Niiharisonshi-shiryoushuu-dai4hen. Ibaraki: Takahashi-Insatsu; 1985. (in Japanese) | Nagai_Hongou_Yukiiri_Kamisaya | 36.16736 | 140.17486 | Ridge |  | 1870 |
| Niiharisonshi-hennsannjimukyoku. Niiharisonshi-shiryoushuu-dai4hen. Ibaraki: Takahashi-Insatsu; 1985. (in Japanese) | Nagai_Hongou_Yukiiri_Kamisaya | 36.16736 | 140.17486 | Road |  | 1870 |
| Niiharisonshi-hennsannjimukyoku. Niiharisonshi-shiryoushuu-dai4hen. Ibaraki: Takahashi-Insatsu; 1985. (in Japanese) | Nagai_Hongou_Yukiiri_Kamisaya | 36.16736 | 140.17486 | Mound |  | 1870 |
| Niiharisonshi-hennsannjimukyoku. Niiharisonshi-shiryoushuu-dai4hen. Ibaraki: Takahashi-Insatsu; 1985. (in Japanese) | Nagai_Hongou_Yukiiri_Kamisaya | 36.16736 | 140.17486 | Pile |  | 1870 |
| Torideshishi-hennsanniinkai. Torideshishi-kinseishiryou3. Tokyo: Seikousha; 1991. (in Japanese) | Toride_Daishuku | 35.897361 | 140.064595 | Shrine gate |  | 1812 |
| Torideshishi-hennsanniinkai. Torideshishi-kinseishiryou3. Tokyo: Seikousha; 1991. (in Japanese) | Toride_Daishuku | 35.897361 | 140.064595 | Buddhist invocation hall |  | 1812 |
| Torideshishi-hennsanniinkai. Torideshishi-kinseishiryou3. Tokyo: Seikousha; 1991. (in Japanese) | Toride_Daishuku | 35.897361 | 140.064595 | Bonten (strips of white paper hung from branches) |  | 1814 |
| Torideshishi-hennsanniinkai. Torideshishi-kinseishiryou3. Tokyo: Seikousha; 1991. (in Japanese) | Toride_Daishuku | 35.897361 | 140.064595 | Pile |  | 1814 |
| Torideshishi-hennsanniinkai. Torideshishi-kinseishiryou3. Tokyo: Seikousha; 1991. (in Japanese) | Toride_Daishuku | 35.897361 | 140.064595 | Road |  | 1814 |
| Torideshishi-hennsanniinkai. Torideshishi-kinseishiryou3. Tokyo: Seikousha; 1991. (in Japanese) | Toride_Daishuku | 35.897361 | 140.064595 | Ditch |  | 1814 |
| Torideshishi-hennsanniinkai. Torideshishi-kinseishiryou3. Tokyo: Seikousha; 1991. (in Japanese) | Toride_Daishuku | 35.897361 | 140.064595 | Foundation stone |  | 1814 |
| Tamatsukurishishi-hennsanniinkai. Tamatsukurichoushi. Tokyo: Seikousha; 1985. (in Japanese) | Onuki_Tega_Tamatsukuri | 36.104292 | 140.452727 | Road |  | 1649 |
| Tamatsukurishishi-hennsanniinkai. Tamatsukurichoushi. Tokyo: Seikousha; 1985. (in Japanese) | Onuki_Tega_Tamatsukuri | 36.104292 | 140.452727 | Bank |  | 1649 |
| Iwaishishi-hennsanniinkai. Iwaishishi-shiryou-kinseihen2. Tokyo: Daiichihouki; 1995. (in Japanese) | Sugao_Houshito_Yahagi | 35.990922 | 139.922622 | Road |  | 1778 |
| Sashimachoushi-hennsanniinkai. Sashimachoushi-shiryouhen-kinsei. Tokyo: Seikousha; 1995. (in Japanese) | Kutsukake_Oigo | 36.114343 | 139.891505 | Road |  | 1836 |
| Sashimachoushi-hennsanniinkai. Sashimachoushi-shiryouhen-kinsei. Tokyo: Seikousha; 1995. (in Japanese) | Kutsukake_Oigo | 36.114343 | 139.891505 | Pile |  | 1836 |
| Sashimachoushi-hennsanniinkai. Sashimachoushi-shiryouhen-kinsei. Tokyo: Seikousha; 1995. (in Japanese) | Sakasai | 36.14416 | 139.852039 | Pile |  | 1844 |
| Hokotachoushi-hennsanniinkai. Hokotachoushi-tsuushihen-jyoukan. Tokyo: Gyousei; 2000. (in Japanese) | Kariyado_Aoyagi | 36.161201 | 140.159182 | Road |  | 1626 |
| Hokotachoushi-hennsanniinkai. Hokotachoushi-tsuushihen-jyoukan. Tokyo: Gyousei; 2000. (in Japanese) | Habara_Tamatsukuri_the other 24 villages | 36.137914 | 140.454552 | Mound |  | 1679 |
| Hokotachoushi-hennsanniinkai. Hokotachoushi-kinseishiryouII. Ibaraki: Katsura-Insatsu; 1998. (in Japanese) | Kariyado_Serizawa | 36.152486 | 140.453289 | Road |  | 1652 |
| Hokotachoushi-hennsanniinkai. Hokotachoushi-kinseishiryouII. Ibaraki: Katsura-Insatsu; 1998. (in Japanese) | Kariyado_Serizawa | 36.152486 | 140.453289 | Bank |  | 1652 |
| Hokotachoushi-hennsanniinkai. Hokotachoushi-kinseishiryouII. Ibaraki: Katsura-Insatsu; 1998. (in Japanese) | Kariyado_Serizawa | 36.152486 | 140.453289 | Pile |  | 1652 |
| Hokotachoushi-hennsanniinkai. Hokotachoushi-kinseishiryouII. Ibaraki: Katsura-Insatsu; 1998. (in Japanese) | Touma_Sakado | 36.169558 | 140.48549 | Road |  | 1668 |
| Hokotachoushi-hennsanniinkai. Hokotachoushi-kinseishiryouII. Ibaraki: Katsura-Insatsu; 1998. (in Japanese) | Touma_Sakado | 36.169558 | 140.48549 | River |  | 1668 |
| Hokotachoushi-hennsanniinkai. Hokotachoushi-kinseishiryouII. Ibaraki: Katsura-Insatsu; 1998. (in Japanese) | Kariyado_Habara | 36.145638 | 140.472339 | Road |  | 1670 |
| Hokotachoushi-hennsanniinkai. Hokotachoushi-kinseishiryouII. Ibaraki: Katsura-Insatsu; 1998. (in Japanese) | Kariyado_Habara | 36.145638 | 140.472339 | Bank |  | 1670 |
| Hokotachoushi-hennsanniinkai. Hokotachoushi-kinseishiryouII. Ibaraki: Katsura-Insatsu; 1998. (in Japanese) | Kariyado_Habara | 36.145638 | 140.472339 | Mound |  | 1670 |
| Uchiharachoushi-hennsanniinkai. Uchiharachoushi-tsuushihen. Tokyo: Seikousha; 1996. (in Japanese) | Ushibushi_Ikenobe | 36.424168 | 140.355716 | Road |  | 1872 |
| Uchiharachoushi-hennsanniinkai. Uchiharachoushi-tsuushihen. Tokyo: Seikousha; 1996. (in Japanese) | Miyu_Kobayashi | 36.363588 | 140.135857 | Mound |  | 1813 |
| Uchiharachoushi-hennsanniinkai. Uchiharachoushi-tsuushihen. Tokyo: Seikousha; 1996. (in Japanese) | Miyu_Kobayashi | 36.363588 | 140.135857 | Trench |  | 1813 |
| Uchiharachoushi-hennsanniinkai. Uchiharachoushi-tsuushihen. Tokyo: Seikousha; 1996. (in Japanese) | Miyu_Kobayashi | 36.363588 | 140.135857 | Upland corner |  | 1813 |
| Uchiharachoushi-hennsanniinkai. Uchiharachoushi-tsuushihen. Tokyo: Seikousha; 1996. (in Japanese) | Miyu_Kobayashi | 36.363588 | 140.135857 | Valley |  | 1813 |
| Uchiharachoushi-hennsanniinkai. Uchiharachoushi-tsuushihen. Tokyo: Seikousha; 1996. (in Japanese) | Miyu_Kobayashi | 36.363588 | 140.135857 | Mound |  | 1869 |
| Uchiharachoushi-hennsanniinkai. Uchiharachoushi-tsuushihen. Tokyo: Seikousha; 1996. (in Japanese) | Kobayashi_Uchihara | 36.363242 | 140.346479 | Mound |  | 1680 |
| Uchiharachoushi-hennsanniinkai. Uchiharachoushi-tsuushihen. Tokyo: Seikousha; 1996. (in Japanese) | Koibuchi_Kobayashi | 36.354927 | 140.360291 | Mound |  | 1668 |
| Uchiharachoushi-hennsanniinkai. Uchiharachoushi-tsuushihen. Tokyo: Seikousha; 1996. (in Japanese) | Koibuchi_Kobayashi | 36.354927 | 140.360291 | Pile |  | 1668 |
| Uchiharachoushi-hennsanniinkai. Uchiharachoushi-tsuushihen. Tokyo: Seikousha; 1996. (in Japanese) | Sugisaki_Obara | 36.374389 | 140.336845 | Mound |  | 1689 |
| Mihosonshi-hennsanniinkai. Mihosonshi-Mihomura-tanjyou-40shuunennkinen. Chiba: Ronshoboushuppan; 1995. (in Japanese) | Jinai_Ukeryou_Kihara | 36.012344 | 140.315463 | Mound |  | 1638 |
| Mihosonshi-hennsanniinkai. Mihosonshi-Mihomura-tanjyou-40shuunennkinen. Chiba: Ronshoboushuppan; 1995. (in Japanese) | Jinai_Ukeryou_Kihara | 36.012344 | 140.315463 | Ridge |  | 1638 |
| Mihosonshi-hennsanniinkai. Mihosonshi-Mihomura-tanjyou-40shuunennkinen. Chiba: Ronshoboushuppan; 1995. (in Japanese) | Jinai_Ukeryou_Kihara | 36.012344 | 140.315463 | Valley |  | 1638 |
| Yukishishi−hennsanniinkai. Yukishishi-dai5kan-kinseitsuushihen. Tokyo: Seikousha; 1983. (in Japanese) | Ooki_Kamiyamakawa | 36.255896 | 139.858355 | Mound |  | 1655 |
| Ryugasakishishi-hennsanniinkai. Ryugasakishishi-kinseishiryouhen1. Tokyo: Gyousei; 1990. (in Japanese) | Nareuma_Ryugasaki | 35.926755 | 140.188851 | Road |  | 1687 |
